# Empagliflozin prevents heart failure through inhibition of the NHE1-NO pathway, independent of SGLT2

**DOI:** 10.1007/s00395-024-01067-9

**Published:** 2024-07-24

**Authors:** Sha Chen, Qian Wang, Diane Bakker, Xin Hu, Liping Zhang, Ingeborg van der Made, Anna M. Tebbens, Csenger Kovácsházi, Zoltán Giricz, Gábor B. Brenner, Peter Ferdinandy, Gert Schaart, Anne Gemmink, Matthijs K. C. Hesselink, Mathilde R. Rivaud, Michael P. Pieper, Markus W. Hollmann, Nina C. Weber, Jean-Luc Balligand, Esther E. Creemers, Ruben Coronel, Coert J. Zuurbier

**Affiliations:** 1 Laboratory of Experimental Intensive Care and Anesthesiology (L.E.I.C.A.), Department of Anesthesiology, Amsterdam Cardiovascular Sciences, Amsterdam UMC, University of Amsterdam, Meibergdreef 9, 1105 AZ Amsterdam, The Netherlands; 2 Dalton Cardiovascular Research Center, University of Missouri, Columbia, USA; 3 Department of Experimental Cardiology, Amsterdam Cardiovascular Sciences, Amsterdam UMC, University of Amsterdam, Amsterdam, The Netherlands; 4 HUN-REN–SU System Pharmacology Research Group, Department of Pharmacology and Pharmacotherapy, Semmelweis University, 1089 Budapest, Hungary; 5 Pharmahungary Group, 6722 Szeged, Hungary; 6 Department of Nutrition and Movement Sciences, NUTRIM School for Nutrition and Translational Research in Metabolism, Maastricht University Medical Centre+, Maastricht, The Netherlands; 7 CardioMetabolic Diseases Research, Boehringer Ingelheim Pharma GmbH & Co KG, Biberach an der Riss, Germany; 8 Pole of Pharmacology and Therapeutics, Institut de Recherche Experimentale et Clinique (IREC) and Cliniques Universitaires Saint-Luc, Université Catholique de Louvain (UCLouvain), Brussels, Belgium

**Keywords:** Heart failure, NHE1, SGLT2 inhibitors, Oxidative stress, Nitric oxide, Diastolic dysfunction

## Abstract

Sodium glucose cotransporter 2 inhibitors (SGLT2i) constitute the only medication class that consistently prevents or attenuates human heart failure (HF) independent of ejection fraction. We have suggested earlier that the protective mechanisms of the SGLT2i Empagliflozin (EMPA) are mediated through reductions in the sodium hydrogen exchanger 1 (NHE1)-nitric oxide (NO) pathway, independent of SGLT2. Here, we examined the role of SGLT2, NHE1 and NO in a murine TAC/DOCA model of HF. SGLT2 knockout mice only showed attenuated systolic dysfunction without having an effect on other signs of HF. EMPA protected against systolic and diastolic dysfunction, hypertrophy, fibrosis, increased Nppa/Nppb mRNA expression and lung/liver edema. In addition, EMPA prevented increases in oxidative stress, sodium calcium exchanger expression and calcium/calmodulin-dependent protein kinase II activation to an equal degree in WT and SGLT2 KO animals. In particular, while NHE1 activity was increased in isolated cardiomyocytes from untreated HF, EMPA treatment prevented this. Since SGLT2 is not required for the protective effects of EMPA, the pathway between NHE1 and NO was further explored in SGLT2 KO animals. In vivo treatment with the specific NHE1-inhibitor Cariporide mimicked the protection by EMPA, without additional protection by EMPA. On the other hand, in vivo inhibition of NOS with L-NAME deteriorated HF and prevented protection by EMPA. In conclusion, the data support that the beneficial effects of EMPA are mediated through the NHE1-NO pathway in TAC/DOCA-induced heart failure and not through SGLT2 inhibition.

**Supplementary Information:**

The online version contains supplementary material available at 10.1007/s00395-024-01067-9.

## Introduction

Sodium glucose cotransporter-2 inhibitors (SGLT2i) now belong to first line armamentarium for the treatment of chronic heart failure (HF) due to their cardiovascular protective effects. Two mechanisms have been suggested. First, SGLT2i inhibit the target-protein SGLT2 in the kidney, facilitating improvements in hemodynamics, volume loading, natriuresis and whole-body nutrient homeostasis. Second, SGLT2i directly target the heart, thereby improving cardiac ion homeostasis, nutrient- and redox signaling, inflammation and remodelling [11, 50]. The heart is largely devoid of SGLT2, and previous work has demonstrated that the SGLT2i empagliflozin (EMPA) protects against myocardial infarction, independent of SGLT2 [14, 48]. It has also been shown that the effects of SGLT2i in the heart are mediated, at least partly, through direct inhibition of cellular sodium loaders in the plasma membrane of cardiomyocytes, such as the sodium hydrogen exchanger 1 (NHE1) [4, 30, 62, 63, 75], the sodium-calcium exchanger (NCX) [37, 68] or the Nav1.5 sodium channel [26, 37, 45, 53]. The resulting lowering of intracellular sodium and calcium through inhibition of these sodium regulating proteins can drive reductions in oxidative stress and Calcium/calmodulin-stimulated protein kinase II (CaMKII) signalling [11, 18] through Ca^2+^-regulated protein kinase C (PKC)-nicotinamide adenine dinucleotide phosphate oxidases (NOX) inhibition [38, 65] and the NHE1/NCX/reactive oxygen species (ROS)/CamKII/Nitric oxide (NO) pathway [18]. We have hypothesized previously that NO may be the end-effector of the beneficial cardiovascular effects of SGLT2i [18].

Here, we examined the role for SGLT2, NHE1 and NO in the protective mechanisms of EMPA against HF using wild-type (WT) and SGLT2-knock-out (KO) C57Bl/6N mice (carrying the mitochondrial NNT enzyme) in which HF was induced by combined transverse aortic constriction and implantation of a deoxycorticosterone acetate pellet (TAC/DOCA) as a model system. In this model HF with preserved or mildly reduced ejection fraction (HFpEF, HFmrEF) [43, 44] has previously been reported. The TAC/DOCA insult caused cardiac dysfunction, hypertrophy, lung edema, increased NHE1 activity of isolated cardiomyocytes, elevated NCX expression, increased oxidative and nitrosative stress, and CamKII activation. EMPA was able to reduce all these HF parameters, and EMPA’s protection was equally present in WT and SGLT2 KO mice, demonstrating that protection by EMPA is not through SGLT2 inhibition. We had hypothesized earlier that EMPA protects through attenuation of this interactive NHE1/NCX/oxidative stress/CamKII/NO pathway [11, 18]. Indeed, chronic NHE1 Inhibition in vivo mimicked all EMPA protective effects against HF, and nullified any additional protection by EMPA. Additionally, chronic in vivo inhibition of NO synthesis prevented EMPA to be protective. Thus, our findings support that the SGLT2i EMPA offers protection against HF through inhibition of the NHE1-NO pathway with no role for inhibition of the SGLT2 protein.

## Methods

### Animals

All animal experimental procedures received approval from the Animal Ethics Committee of the Academic Medical Center, Amsterdam, the Netherlands, and were carried out in compliance with the Guide for the Use and Care of Laboratory Animals. SGLT2 global KO mice were generated using CRISPR/Cas9 techniques targeting exon 4 of *Slc5a2*, bred on a C57BL/6N background by hetero x hetero breeding generating male and female wild-type (WT) and KO littermates, as was previously reported [14]. All animals were housed at 22 °C with a 12 h dark cycle with unrestricted access to control chow for a minimum of 14 d before the start of experiments.

### Timeline of all animal experiments

**Fig. 1:**
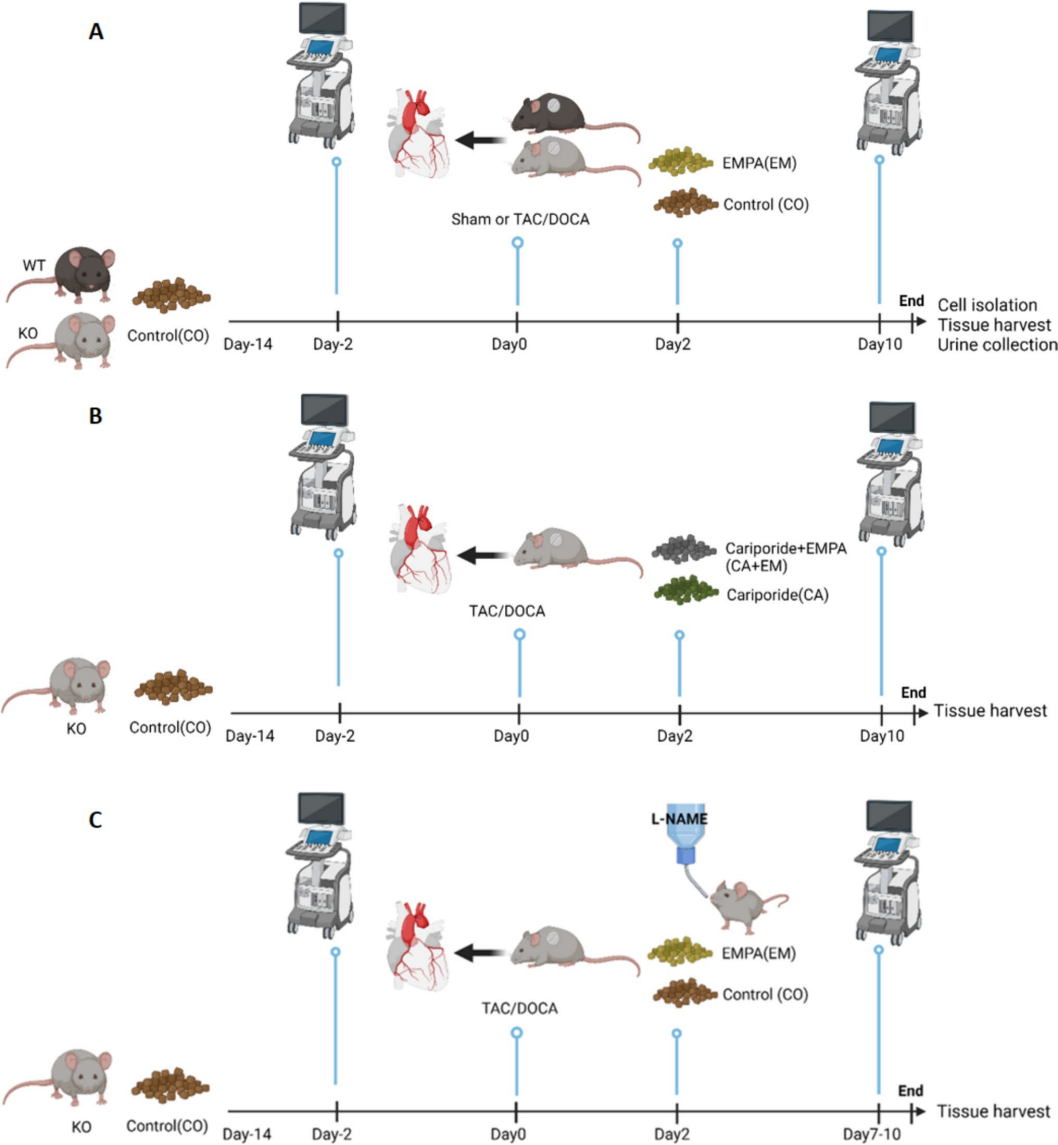
Timeline of interventions. **A** Series #1: Baseline cardiac function was evaluated using ultrasound two days prior surgery to induce HF. Two days post-surgery, oral treatment (CO and EM) [67] was started by switching to treatment-enriched chows. Then, 10 days after Sham or TAC/DOCA surgery, cardiac function was re-evaluated to determine parameters of cardiac HF. Finally, at days 10–12 after start HF surgery, animals were sacrificed. Hearts were used either for cell isolation to perform NHE1 activity assays or immunoblotting. Other organs and urine were collected for further measurements. **B** Series #2: Two days post-surgery, oral treatment (CA and CA + EM) was started by switching to treatment-enriched chows. Then, 10 days after Sham or TAC/DOCA surgery, cardiac function was re-evaluated. Finally, at days 10–12 after start HF surgery, animals were sacrificed. Hearts were used for immunoblotting. Other organs were collected for further measurements. **C** Series #3: Two days post-surgery, oral treatment (CO and EM) was started by switching to treatment-enriched chows and/or L-NAME in drinking water [39]. Then, 7–10 days after Sham or TAC/DOCA surgery, cardiac function was re-evaluated. Finally, at days 8–11 after start HF surgery, animals were sacrificed. Organs were collected for further measurements. *CO* control, *EM* EMPA, *CA* Cariporide, *CA + EM* Cariporide + EMPA, *L-NAME* L-nitro-arginine-methyl-esther, *TAC/DOCA* Transverse aortic constriction and implantation of a deoxycorticosterone acetate pellet. (Created with BioRender.com)

Baseline cardiac function was evaluated using ultrasound two days prior to surgery to induce HF. Body weight was recorded once a day following Sham or TAC/DOCA surgery. Two days post-surgery, oral treatment (series #1 and #3:control, EMPA; series #2: Cariporide, and Cariporide + EMPA), was started by switching to treatment-enriched chows and/or L-nitro-arginine-methyl-esther (L-NAME, series #3) in drinking water [39] (see below Experimental groups). Cardiac function was re-evaluated to determine parameters of cardiac HF 10 days after Sham or TAC/DOCA surgery. Finally, at days 10–12 (series #1 and #2) or 7–10 (series #3) after HF surgery, animals were sacrificed. Hearts were used either for cell isolation to perform NHE1 activity assays or for immunoblotting. Urine and tissues were collected (Fig. 1A, C).

### Experimental groups

Six different groups of both male (M) and female (F) mice were studied: (1) Sham WT with control chow (SH-WT-CO) (n = 6, 3 M, 3F), (2) Sham KO with control chow (SH-KO-CO)) (n = 7, 3 M, 4F), (3) TAC/DOCA WT with control chow (HF-WT-CO)) (n = 12, 6 M, 6F), (4) TAC/DOCA KO with control chow (HF-KO-CO)) (n = 14, 6 M, 8F), (5) TAC/DOCA WT with EMPA chow (HF-WT-EM) (n = 6, 3 M, 3F), and (6) TAC/DOCA KO with EMPA chow (HF-KO-EM)) (n = 9, 4 M, 5F). Chow was enriched with 300 mg/kg EMPA in regular rodent diet (in%: 19 protein, 3.3 fat, 4.9 fiber, 6.4 ash, 36.5 starch, 4.7 sugar, 12.8 MJ ME/kg; synthesized at Ssniff Spezialdiäten, Soest, Germany) or with control diet as reported previously [67]. EMPA was provided by Boehringer Ingelheim Pharma, Biberach, Germany.

From series #1 it became clear that EMPA’s protective effects against HF were independent of SGLT2. Thus, subsequent series of experiments were performed in SGLT2 KO animals solely, thereby preventing that EMPA effects through SGLT2 on cellular mechanisms were still included. In the second series of experiments (series #2), the NHE1 specific inhibitor Cariporide (Sanofi, Germany) was added to the regular rodent chow at a dosage of 600 mg/kg chow. This dose previously resulted in plasma concentrations of 2.5 µM ensuring full inhibition of NHE1 in vivo [19, 40]. Two groups were examined and compared with the previously obtained group of HF-KO-CO, i.e. (1) HF-KO-CO-CA (n = 9, 4 M, 5F), and (2) HF-KO-EM-CA (n = 7, 3 M, 4F).

In the third series of experiments (series #3), mice were treated with the nonspecific nitric oxide synthase (NOS) inhibitor, L-NAME, at a concentration of 2 mg/ml in the drinking water [6, 39]. To test whether the protection provided by EMPA was NO-mediated, two groups were examined and compared with the previously obtained control group of HF-KO-CO, i.e. (1) HF-KO-CO-NA (NA:L-NAME treated; n = 11, 7 M, 4F), and (2) HF-KO-EM-NA (n = 11, 6 M, 5F).

### TAC surgery + DOCA pellet implantation

For the induction of HF, mice underwent TAC combined with the insertion of a DOCA pellet (50 mg/pellet, 35-day release, Innovative Research America) in the neck area [43]. Mice underwent anesthesia induction with 4% isoflurane, followed by maintenance with 2% isoflurane at a flow rate of 1 l/min of 30–40% O_2_. Mice were intubated using a 20-gauge polyethylene catheter and placed on a heating pad to sustain a body temperature of 37 °C and connected to a mouse ventilator. The thoracic cavity was reached via a small incision made within the second intercostal space. Using a 7–0 silk suture, we encircled the aorta between the right innominate and left common carotid artery. Constriction of the transverse aorta was achieved by securing it against a 27G needle, which was then quickly extracted. Sham animals received the same procedural steps, with the exception of aortic constriction [55]. A DOCA or placebo pellet was implanted under the skin in the neck region of the animal [43]. Animals were allowed to recover in a ThermoCare warmer at 37 °C. Carprofen (0.06 mg/ml) was put into drinking water 2 days before surgery. Buprenorphine (Buprenex; 0.075 mg/kg) was administrated i.p. before start surgery, and subcutaneously administered together with Carprofen (5 mg/kg) twice a day for 3 days after surgery.

### Echocardiography

Mice were anesthetized with 2.5–3% isoflurane for induction and 1.0–1.5% isoflurane for maintenance at a flow rate of 1 l/min of 30–40% O_2_. Mice were placed on a heating pad and ECG was recorded. In vivo cardiac function was evaluated through transthoracic echocardiography, utilizing a Vevo 3100 high-resolution imaging system equipped with a 40-MHz transducer (VisualSonics, Toronto, Ontario, Canada). Images from parasternal long axis and short axis view and 4-chamber view were acquired. Full systolic and diastolic parameters were assessed both 2 days before and 10 days after TAC/DOCA surgery. Measurements were averaged at least three cardiac cycles. The following parameters were assessed: The ratio of early velocity of diastolic transmitral valve blood flow to late velocity of diastolic transmitral valve blood flow (E/A ratio), the ratio of early velocity of diastolic transmitral valve blood flow to early diastolic mitral annulus velocity (E/e' ratio), Left atria internal diameter at end diastole (LAIDd, mm), Left ventricle mass (LV mass Cor, mg), Ejection fraction (%EF), Fractional shortening (%FS), Heart rate (HR, bpm), Stroke volume (SV, ml), Cardiac output (CO, ml/min) and aortic root diameter. Echocardiography images were analyzed by the echocardiographist (SC) who was blinded to the group assignment of the mice. Echo images were selected at random for verification analysis by an external observer who was also blinded to group assignment.

### Post-experimental tissue collection and analysis of hearts

At day 10–12 after TAC surgery, urine was collected from each animal in their cage, and mice were anesthetized by i.p. sodium pentobarbital (95 mg/kg) and simultaneously injected with the anti-coagulant heparin (15 IU). Once the withdrawal reflexes to hind limb toe pinch were no longer evident, mice underwent intratracheal mechanical ventilation with a mixture of 50% O_2_ and 50% N_2_. For series #1 hearts were isolated either for cardiomyocyte isolation or for analysis of immunoblotting and fibrosis, whereas hearts from series #2 and #3 were weighted and stored at − 80 °C. From all animals, lung and liver were harvested, weighed, and dried for 2 days at 37 °C for determination of wet/dry weight ratio (edema). For analysis of fibrosis and immunoblotting (series #1 and #2 hearts), left and right atria and right ventricle were quickly separated, weighed and dropped in liquid nitrogen for storage at − 80 °C. Left ventricle (LV) free wall and septum were separated, weighed, and septum dropped in liquid nitrogen to store at − 80 °C. LV free wall was cut into three equal parts: base, medial and apex. Base and apex were rapidly frozen in liquid nitrogen and stored at − 80 °C. The medial part was put on a drop of Tissue-Tek^®^ O.C.T. Compound (Sakura, 4583), quickly submerged into liquid nitrogen-precooled Isopentane (Sigma/Merck, 277,258) for 1 min and then stored at − 80 °C for Picrosirius red staining to determine percentage of fibrosis. Base and apex of LV free wall were used for Western blot.

### Cardiomyocyte isolation from left ventricle of murine hearts

For series #1 hearts, approximately half of the hearts were allocated for cardiomyocyte isolation. Adult mouse ventricular myocytes were obtained through enzymatic dissociation following standard protocols. Briefly, after 10–12 days of Sham or TAC/DOCA surgery, mice were anesthetized using intraperitoneal administration of sodium pentobarbital (95 mg/kg), followed by intraperitoneal injection of heparin (15 IU). Additional pentobarbital was administered intramuscularly as needed to achieve appropriate anesthesia depth. After the hind limb withdrawal reflexes to toe pinch ceased to be observable, mice underwent intratracheal ventilation with a mixture of 50% O_2_ and 50% N_2_. Subsequent steps involved chest opening, heart isolation, aortic cannulation using a 22G blunt needle, and connection to a Langendorff setup. Hearts were perfused for 6 min at 37 °C with a solution containing (in mM) 5 HEPES, 140.2 NaCl, 5.4 KCl, 1.8 CaCl_2_, 1 MgCl_2_ and 5.5 glucose (pH maintained at 7.4) to remove remaining blood from the heart. Next, perfusion was then switched to a low-calcium solution comprising (in mM) 5 HEPES, 140.2 NaCl, 5.4 KCl, 0.009 CaCl_2_, 1 MgCl_2_, 5.5 glucose and 0.01 Creatine monohydrate (pH maintained at 7.4). After 10 min, the heart underwent 8 min perfusion with same solution at a constant flow in a recirculating manner with cell dissociation enzymes added (19U/ml Elastase, Worthington Biochemical and 2U/ml LiberaseTM Research Grade, Roche Chemicals). The LVs were subsequently sectioned into small pieces and delicately minced using a Pasteur pipette. Gradual restoration of calcium concentration to normal levels was ensued. Isolated cardiomyocytes were subsequently used for NHE1 activity measurements on the same day of isolation.

### NHE1 activity measurement

NHE1 activity was measured as previously described [13]. To assess intracellular pH levels, isolated rod-shaped cardiomyocytes were incubated with 10 μM SNARF-AM (C1270, Thermofisher) for 30 min in a 37 °C water bath within a HEPES solution (mM): HEPES 17.0, NaHCO_3_ 1.0, KHCO_3_ 3.3, KH_2_PO_4_ 1.4, CaCl_2_ 1.3, MgCl_2_ 2.0, NaCl 144, glucose 11.0. SNARF-loaded mice cardiomyocytes were fixed to a poly-D-lysine (0.1 g/l) treated coverslip for NHE1 activity measurement. The experiments were conducted using a temperature-controlled (37 °C) perfusion chamber (height 0.4 mm, diameter 10 mm, volume 30 μl) where the coverslips were positioned. NHE1 activity was determined by recording SNARF-fluorescence (580/640 nm emission; 515 nm excitation) following an NH_4_^+^ pulse. After a 30-s stabilization period, the medium was rapidly (~ 0.1 s) replaced with the same solution now containing 20 mM NH_4_Cl for 10 min, inducing intracellular alkalosis. Subsequently, the NH_4_Cl was swiftly replaced (~ 0.1 s) with a normal solution, resulting in nearly instantaneous intracellular acidosis, followed by a 5 min monitoring period for recovery from acidosis. Rod-shaped cardiomyocytes underwent field stimulation at a frequency of 2 Hz. Dual-wavelength emission characteristics were assessed while subjected to 100 ms light flashes occurring at a frequency of 1 kHz. The impact of 1 µM EMPA (MedChem Express, Monmouth Junction, NJ, USA) or 0.02% DMSO (D2650, Sigma/Merck) or 10 µM Cariporide (SML1360, Sigma/Merck) on the recovery of acidosis was assessed, and these compounds were present during the NH_4_Cl pulse and the subsequent washout period of NH_4_Cl. The washout period of NH_4_Cl, during which either DMSO, EMPA were present, determined the initial rate of intracellular H^+^ recovery following ammonium washout. This initial rate corresponded to the slope of the linear fit of intracellular H^+^ during the first 100 s of intracellular H^+^ recovery.

### Tissue homogenization

The LV free walls were collected and stored in − 80 °C freezer. Homogenization buffer was made by 0.25 M sucrose and 0.02 M HEPES at pH of 7.4. These tissues were frozen in liquid nitrogen and subsequently smashed mechanically. Then homogenization buffer combined with 1:100 Halt TM Protease and Phosphatase Inhibitor Cocktail (Thermo Fisher Scientific, Waltham, MA, USA) was added into liquid nitrogen-precooled tissues and then sonicated for 15 s. Next, 0.5% Triton-100X was introduced and incubated on ice for 10 min, followed by centrifugation for 10 min at 15000 rpm. Finally, aliquots were made.

### Immunoblotting

Lowry determination was used to detect protein concentration. Following sample buffer was introduced, samples are put on heat block for 5 min at 95 °C. After a quick centrifugation, samples are loaded into 4–12% Bis–Tris precast SDS–polyacrylamide gel (#3450125, Bio-Rad) for electrophoresis. Next, protein was transferred to olyvinylidene fluoride membrane (IPFL00010, Millipore) and Ponceau S solution (0.1% Ponceau S in 1% acetic acid) was used to determine total protein. Image J software (Version 1.54d, NIH, USA) was used for analysis of total protein. Ponceau S solution was removed by Milli Q and Methanol was used to rinse the membrane. Membrane was washed by Phosphate buffered saline with 0.1% Tween 20 (P2287, Sigma) for 3 times with 5 min each time. Then membrane was blocked by blocking buffer (927–70,001, LI-COR) for 1 h at room temperature. Primary antibodies was employed overnight at 4 ºC for mice hearts: NHE1 (1:100, MAB3140, Millipore EMD), 4-Hydroxynonenal (4-HNE), (1:250, ab46545, Abcam); NCX1 (1:1000, #79350, CST); 3-Nitrotyrosine (3-NT, 1:250, ab61392, Abcam); Superoxide dismutase 1 (SOD1, 1:5000, ab51254, Abcam), CaMK2 beta gamma delta (phospho T287), (1:500, ab182647, Abcam); SGLT2 (1:1000, 28683–1, Proteintech). Primary antibodies was employed overnight at 4 ºC for rat H9c2 cells: NHE1 (1:1000, ab67313, Abcam). NCX1 (1:1000, #79350, CST). After washing the membrane 3 times with 5 min each time, secondary antibodies were used to incubate the membrane for 1 h at room temperature. The membrane was washed 3 times with 5 min each time. Odyssey CLx Imager (LI-COR Biosciences) and Image Studio™ Software (Version 5.1, LI-COR Bioscience) were used to scan and analyze membrane. The membranes are put in the Supplementary Fig. 2.

### Reverse transcription-quantitative polymerase chain reaction (RT-qPCR)

Total RNA from mice left ventricle free wall and H9c2 cell line were isolated using TriPure™ Isolation Reagent (11,667,165,001, Sigma/Merck). Transcriptor First Strand cDNA Synthesis Kit (4,897,030,001, Roche) and LightCycler^®^ 480 SYBR Green I Master (4,707,516,001, Roche) were used for reverse transcription and amplification respectively. 2^−ΔΔCt^ method was used for gene expression analysis. The primers for mice left ventricle free wall were as follows: ANP: forward: 5′-CACAGATCTGATGGATTTCAAGA-3′; reverse: 5′-CCTCATCTTCTACCGGCATC-3′; BNP: forward: 5′-GTCCAGCAGAGACCTCAAAA-3′; reverse: 5′-AGGCAGAGTCAGAAACTGGA-3′; GAPDH: forward: 5′-TCGGTGTGAACGGATTTGGC-3′; reverse: 5′-TCCCATTCTCGGCCTTGACT-3′; The primers for H9c2 cell line were as follows: BNP: For 5′AGGAGAGACTTCGAAATTCCAAGA3′; Rev 5′CTAAAACAACCTCAGCCCGTCA. GAPDH: For 5′ TCGGTGTGAACGGATTTGGC3′; Rev 5′TCCCATTCTCGGCCTTGACT3′.

### Picro Sirius Red staining

Picro Sirius red staining kit (ab150681, Abcam) was used to determine myocardial fibrosis in cardiac sections (10 µm). Briefly, frozen cryosections were air-dried for 15 min and then incubated with the Picro-Sirius red stain for 60 min. Sections were washed quickly two times with 0.5% acetic acid solution and thereafter with 100% ethanol. Sections were mounted in limonene. Three random fields were collected from each section from each mouse heart under a Nikon E800 light microscope. Images were analyzed using Image J.

### Cell culture

H9c2 rat cardiomyoblast cells were obtained from ATCC (American Type Culture Collection, Manassas, Virginia, USA) and cultured in high glucose (4500 mg/l) Dulbecco’s modified Eagle’s medium (DMEM, D5796, Sigma) supplemented with 10% Fetal Calf Serum (FCS) and 100 U/ml of Penicillin/Streptomycin. Cells were obtained by centrifugation (5 min, 1000 rpm) and subsequently re-suspended in plating medium. Cells were grown in a humidified incubator with an atmosphere of 95% O_2_, 5% CO_2_. Stock cultures were passaged at 2- to 3-day intervals.

To examine the role of NCX in cell hypertrophy we applied the NCX inhibitor SEA0400 using the following protocol. Hypertrophy was induced by treating the cells with 100 μM phenylephrine (P) (Sigma, #P1240000) for 48 h. Different cell treatments were examined and compared: The vehicle (V) group was subjected to DMSO (0.02%). The P group was given 100 µM P and 0.02% DMSO. The P + EMPA (P + E) group was given 100 µM P and 1 µM EMPA (MedChem Express, # HY-15409). The P + SEA0400 (P + S) group was given 100 µM P and 1 µM SEA0400 (MedChem Express, # HY-15515). The P + EMPA + SEA0400 (P + E + S) group was given 100 µM P, 1 µM EMPA and 1 µM SEA0400.

### Small interfering RNA (siRNA) transfection to knockdown NHE1

The knockdown of NHE1 was achieved using 100 nM NHE1 rat siRNA Oligo Duplex (AM16708, Thermo Fisher). 100 nM scrambled siRNA (4,390,843, Thermo Fisher) was used as negative control. H9c2 cells were seeded into 6-well plates at a density of 100,000 cells per well to reach a confluence of 50–70% after 1 days. Before transfections, cells were starved with medium containing 2% FBS for 12 h siRNA Oligo Duplexes or scrambled siRNA were mixed together with 50 nM Lipofectamine 3000 (L3000008, Thermo Fisher) and incubated for 20 min. The mixture was added onto cells and incubated for 24 h with antibiotic-free reduced serum medium (31,985,070, Thermo Fisher). Only for cell surface area measurement, cells were then seeded at a density of 5000 cells per well in 6-well plates (n = 5 independent experiments per group). For immunoblot analysis and RNA extraction, transfected cells were directly used for subsequent treatments: P + scRNA (P + scRNA) and P + siRNA (P + siRNA) groups were treated with 100 µM P. P + EMPA + scRNA (P + E + scRNA) and P + EMPA + siRNA (P + E + siRNA) groups were treated with 100 µM PE and 1 µM EMPA. For BNP analysis, n = 4 independent experiments per group were examined.

### Determination of cell surface area

For analysis of morphometric hypertrophy, cell size was determined using an inverted microscope (20 ×). Cell surface area analysis was performed using Image J software. A minimum of 4 images per group were examined and data is normalized to V group in order to facilitate comparison of relative cell surface area across treatments.

#### Plasma levels of Empagliflozin

To validate that 300 mg EMPA per kg chow resulted in clinically relevant plasma levels of EMPA in our study, as was previously reported by an internal report of Boehringer Ingelheim for different studies, animal were subjected to the TAC/DOCA insult, treated with the EMPA-enriched chow and sacrificed at 6–7 days after the TAC/DOCA insult. Animals (n = 6, 1 WT and 5 KO) were anesthetized with 1.5% isoflurane, mechanical ventilated and blood sampled from the cannulated artery carotid between 9 and 11 AM. Plasma was obtained by centrifugation and stored at – 20 °C for further analysis of EMPA concentrations by Eurofins ADME BIOANALYSES (project 24-0241, Vergèze, France).

### Statistical analysis

Statistical analyses were performed using GraphPad Prism 9.3.1 (GraphPad Software, Inc., La Jolla, CA, USA). Data are presented as mean ± standard deviation (SD). All data were tested for normality distribution by Shapiro–Wilk test. For the data which has two or more than two groups non-normally distributed, Mann–Whitney U test or Kruskal–Wallis with Dunn's test were used for two groups comparisons or three groups comparisons separately. Otherwise, unpaired t-test and one-way ANOVA followed by Holm-Šidák or uncorrected Fisher’s LSD post-hoc test were used for two groups comparisons and three groups comparisons respectively. Log-rank (Mantel-Cox) test was used for survival rate. A p < 0.05 was regarded as statistically significant.

## Results

### SGLT2 knock out does not affect baseline cardiac function or morphology.

**Fig. 2:**
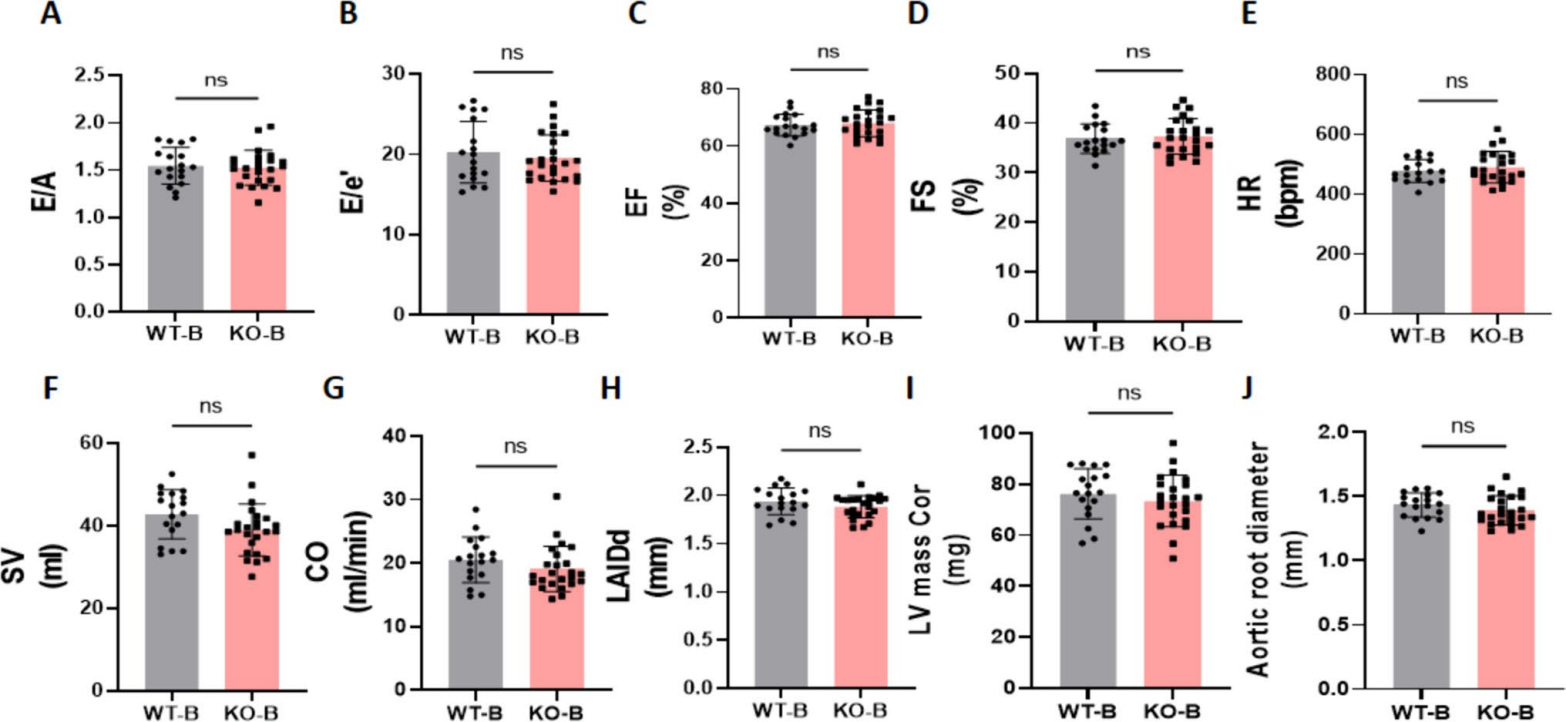
Baseline systolic and diastolic cardiac function in WT and SGLT2 KO mice, before surgery. All of the parameters are measured 2 days before TAC/DOCA surgery. **A**, The ratio of early diastolic mitral inflow velocity to late diastolic mitral inflow velocity (E/A ratio). **B**, The ratio of early diastolic mitral inflow velocity to early diastolic mitral annulus velocity (E/e' ratio). **C**, Ejection fraction (EF). **D**, Fractional shortening (FS). **E**, Heart rate (HR). **F**, Stroke volume (SV). **G**, Cardiac output (CO). **H**, Left atria internal diameter end diastole (LAIDd). **I**, Left ventricle mass (LV mass Cor). **J**, Aortic root diameter. Data are presented as mean ± SD. ns: not significant. WT-B, wild-type baseline (n = 18; 9 M (male), 9F (female)), KO-B, SGLT2 KO baseline (n = 24; 10 M/14F). **A**–**F** and **H**–**J**: unpaired t test; G: Mann–Whitney test. B: Baseline

We first examined whether SGLT2 deletion affected in vivo cardiac function or morphology at baseline (2 days before TAC/DOCA surgery). SGLT2 deficiency did not affect diastolic or systolic cardiac function, as measured by echocardiography (Fig. 2A-1G). Left atria internal diameter end diastole (LAIDd) or left ventricular (LV) mass was also similar between genotypes (Fig. 2H-I). Finally, the aortic diameter (Fig. 2J) at the location of TAC constriction (but without constriction) was not different in SGLT2 knockout mice.

### EMPA ameliorates HF independently of SGLT2, whereas SGLT2 deficiency partly protects against HF-induced systolic dysfunction.

**Fig. 3:**
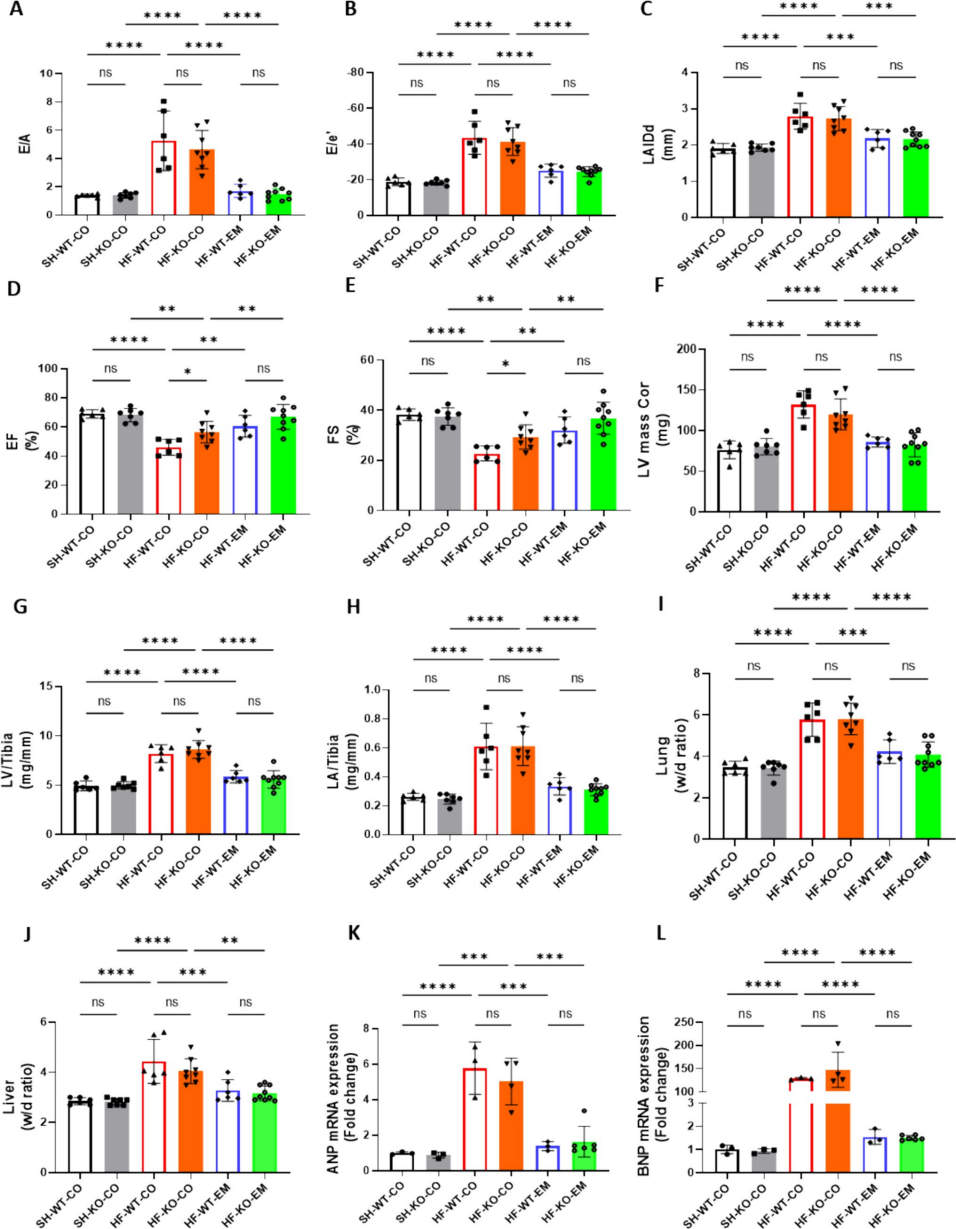
EMPA ameliorated HF independent of SGLT2, whereas SGLT2 deficiency partly protected against HF-induced systolic dysfunction. **A** The ratio of early diastolic mitral inflow velocity to late diastolic mitral inflow velocity (E/A ratio) measured at 10 days after TAC/DOCA surgery. **B** The ratio of early diastolic mitral inflow velocity to early diastolic mitral annulus velocity (E/e' ratio) measured at 10 days after TAC/DOCA surgery. **C** Left atria internal diameter end diastole (LAIDd) measured at 10 days after TAC/DOCA surgery. **D** Ejection fraction (EF) measured at 10 days after TAC/DOCA surgery. **E** Fractional shortening (FS) measured at 10 days after TAC/DOCA surgery. **F** Left ventricle mass (LV mass Cor) measured at 10 days after TAC/DOCA surgery. **G** Left ventricle weight to tibia length(LV/tibia). **H** Left atria weight to tibia length (LA/tibia). **I** Lung wet/dry ratio (Lung w/d ratio). **J**, Liver wet/dry ratio (Liver w/d ratio). **K**, **L** *Nppa* and *Nppb* mRNA expression. Data are presented as mean ± SD. *p < 0.05, **p < 0.01, ***p < 0.001, ****p < 0.0001. ns: not significant. **A**–**J** SH-WT-CO, sham-wild-type-control chow (n = 6; 3 M (male), 3F(female)), SH-KO-CO, sham-SGLT2 KO-control chow (n = 7; 4 M/3F), HF-WT-CO, heart failure-wild-type-control chow (n = 6; 3 M/3F), HF-KO-CO, heart failure-SGLT2 KO-control chow (n = 8; 3 M/5F), HF-WT-EM, heart failure-wild-type-EMPA chow (n = 6; 3 M/3F) and HF-KO-EM, heart failure-SGLT2KO-EMPA chow (n = 9; 4 M/5F). **K**, **L** SH-WT-CO (n = 3; 1 M/2F), SH-KO-CO (n = 3; 1 M/2F), HF-WT-CO (n = 3; 2 M/1F), HF-KO-CO (n = 4; 2 M/2F), HF-WT-EM (n = 3; 2 M/1F) and HF-KO-EM (n = 5; 2 M/3F). **A–L** One-way ANOVA with Holm-Šídák's multiple comparisons test

Glucose measurements demonstrated high glucose in urine of EMPA-treated and SGLT2 KO animals, providing functional confirmation of in vivo EMPA treatment and deletion of SGLT2. (Supplementary Fig. 1A). At baseline, groups were not different with respect to aorta root diameter, whereas 10 days after surgery, diameters were similarly increased among all HF groups, indicating a similar constriction insult (Supplementary Fig. 1B–C). TAC/DOCA caused severe diastolic dysfunction, as reflected by increased E/A, E/e’ and LAIDd (Fig. 3A–C). No statistically significant differences between WT and KO hearts were present, indicating that the absence of SGLT2 did not prevent diastolic dysfunction. EMPA treatment completely prevented diastolic dysfunction independently of the presence of SGLT2. TAC/DOCA also caused mild systolic dysfunction as reflected by a mild reduction in EF and FS. Although not completely normalized and the effect was small, the systolic function was significantly better in the none-treated KO animals as compared to WT. This suggests that SGLT2 may have a subtle role on left ventricular systolic function. In contrast, EMPA treatment fully prevented the mild systolic dysfunction in both WT and KO animals (Fig. 3D–E). In addition, left ventricular and atrial hypertrophy were similarly induced in WT and KO animals, and was prevented with EMPA treatment, independently of SGLT2 (Fig. 3F–H). Similarly, lung and liver edema were induced in this HF model and prevented by EMPA treatment equally in WT and KO mice (Fig. 3I, J). Cardiac *Nppa* and BNP *Nppb* mRNA were also increased following TAC/DOCA, and were not increased with EMPA, independently of SGLT2 (Fig. 3K, L). Finally, left ventricular fibrosis was increased with TAC/DOCA, which was prevented by EMPA treatment in both WT and KO hearts (Supplementary Fig. 1D).Interestingly, EMPA reduced body weight independent of SGLT2 (Supplementary Fig. 1E).

Jointly, these data indicate that the SGLT2i EMPA strongly protects against diastolic and systolic HF in a TAC/DOCA-HF model, independent of the presence of the SGLT2 protein. Whole body deletion of SGLT2 mitigates, but does not fully prevent, HF-induced systolic dysfunction.

### EMPA reduces HF-induced elevations in cardiac NHE1 activity, NCX expression and CamKII activation, independent of SGLT2

**Fig. 4:**
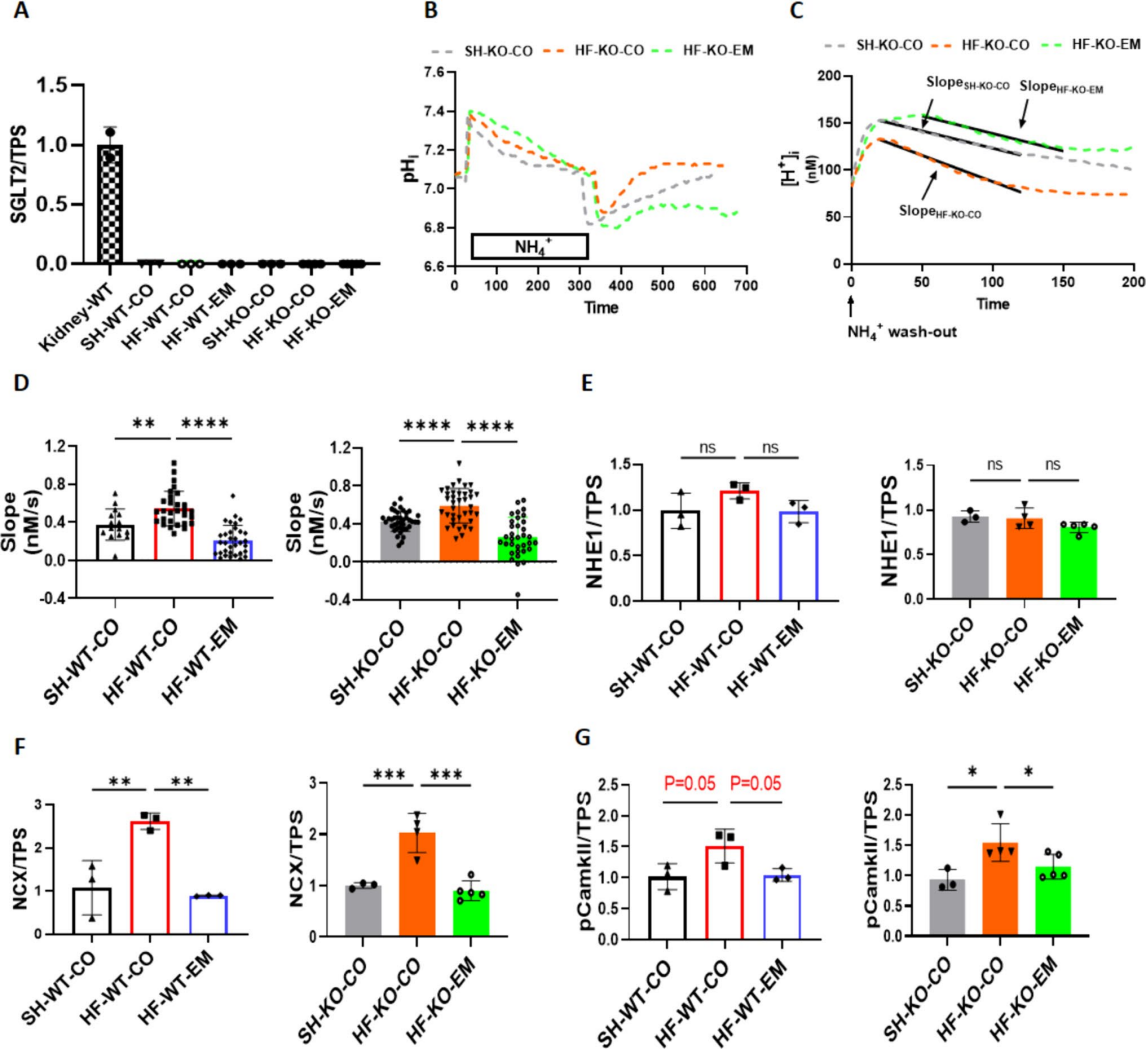
EMPA reduced HF-induced activation of NHE1, NCX expression and CamKII phosphorylation, independently of SGLT2. **A** Sodium glucose cotransporter 2 protein expression/total protein staining (SGLT2/TPS). **B** Typical example of the curve changes of intracellular pH during the whole measurement, showing the slope only for KO mice; **C** Typical example of the curve changes of intracellular [H^+^] during the first 200 s after NH_4_^+^ wash-out, showing the slope only for KO mice. **D** Chronic effects of EMPA on NHE1 activity as reflected by the; **E** Sodium hydrogen exchanger 1 protein expression/total protein staining (NHE1/TPS). **F** sodium calcium exchanger 1 protein expression/total protein staining (NCX/TPS). **G** Phosphorylation of calcium/calmodulin-dependent protein kinase expression/total protein staining (pCamkII/TPS). Data are presented as mean ± SD. *p < 0.05, **p < 0.01, *** p < 0.001, **** p < 0.0001. ns: not significant. **A** and **E–G** and **G-J**: SH-WT-CO (n = 3; 1 M/2F), SH-KO-CO (n = 3; 1 M/2F), HF-WT-CO (n = 3; 2 M/1F), HF-KO-CO (n = 4; 2 M/2F), HF-WT-EM (n = 3; 2 M/1F) and HF-KO-EM (n = 5; 2 M/3F). **B–D** SH-WT-CO (15 cells from 3 mice, 2 M/1F), SH-KO-CO (39 cells from 4 mice, 3 M/1F), HF-WT-CO (29 cells from 3 mice, 1 M/2F), HF-KO-CO (37 cells from 4 mice, 1 M/3F), HF-WT-EM(33 cells from 3 mice, 1 M/2F) and HF-KO-EM (34 cells from 3 mice, 1 M/2F). **D–G** One-way ANOVA with Holm-Šídák's multiple comparisons test

We next studied whether SGLT2 is expressed in the mice hearts with HF. Our data showed that SGLT2 protein expression is absent in the wild-type heart whereas it is highly expressed in kidney, as expected (Fig. 4A, Supplementary Fig. 2A).

In order to measure NHE1 activity, intracellular pH and [H^+^] recovery time curves were recorded from a sudden acid-load induced by an ammonium pre-pulse in isolated cardiac ventricular myocytes. Typical examples of the curve changes of intracellular pH and the derived curve changes of intracellular [H^+^] during the first 200 s after NH_4_^+^ wash-out for each group are shown in Fig. 4B and C, respectively. NHE1 activity was calculated from the slope of the linear fit of the first 100 s of intracellular [H^+^] recovery after intracellular [H^+^] had reached its maximum value. HF increased chronically NHE1 activity in both WT and KO, and treatment by EMPA abolished NHE1 activation in a similar manner for WT and KO left ventricular cells (Fig. 4D). In addition, when EMPA was added to the isolated cardiomyocytes, further inhibition of NHE1 activity was observed, demonstrating direct NHE1 inhibition effects of EMPA (Supplementary Fig. 1F). Note that no changes in NHE1 protein levels were detected with HF or EMPA treatment (Fig. 4E, Supplementary Fig. 2B). Because changes in NHE1 activity result in alterations in Na^+^ or Ca^2+^ homeostasis, we examined NCX protein levels as it is a known responder to changes in these cellular ions [24]. Indeed, HF induced a large increase in NCX protein levels in both WT and KO animals, whereas EMPA prevented the increase, both in the absence and presence of SGLT2 (Fig. 4F, Supplementary Fig. 2C). Furthermore, we checked NCX protein level and the effects of Cariporide treatment. Our data show that the increase of NCX protein level caused by HF is revered and EMPA’s effect on reducing NCX protein expression is lost with Cariporide presence (Supplementary Fig. 1G and Supplementary Fig. 2D). Next, we examined CamKII as one of the suggested downstream effectors of NHE1 inhibition [11, 18]. We observed increased CamKII phosphorylation which indicates increased activation in HF hearts, which was normalized by EMPA treatment, whereby both HF and EMPA effects on CamKII were independent of SGLT2 (Fig. 4G**,** Supplementary Fig. 2E).

### NHE1 inhibition mimics protection against HF by EMPA, with no added protection by EMPA treatment

**Fig. 5:**
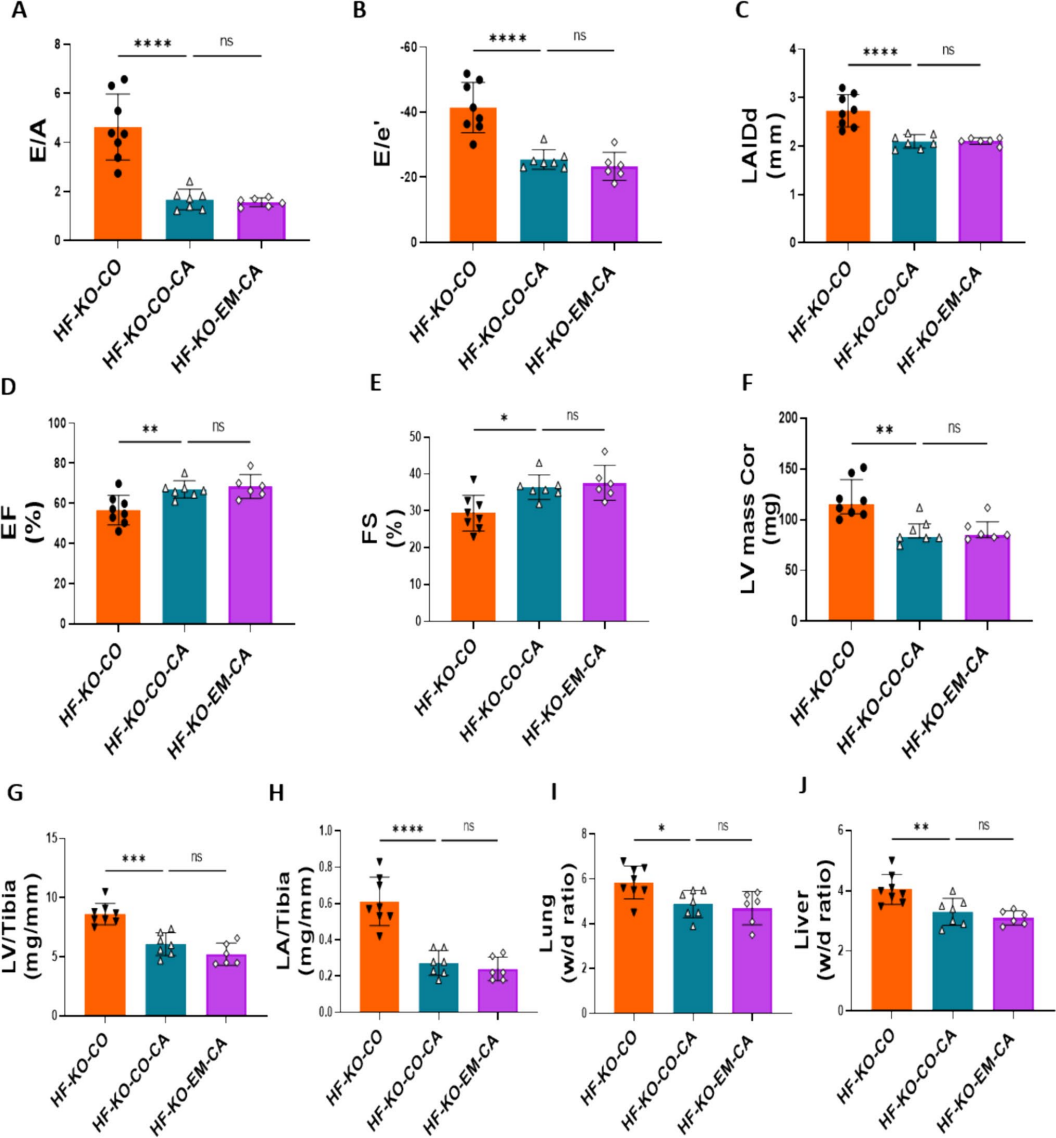
NHE1 inhibitor Cariporide abolished EMPA’s protection against HFmrEF. **A** The ratio of early diastolic mitral inflow velocity to late diastolic mitral inflow velocity (E/A ratio) measured at 10 days after TAC/DOCA surgery. **B** The ratio of early diastolic mitral inflow velocity to early diastolic mitral annulus velocity (E/e' ratio) measured at 10 days after TAC/DOCA surgery. **C** Left atria internal diameter end diastole (LAIDd) measured at 10 days after TAC/DOCA surgery. **D** Ejection fraction (EF) measured at 10 days after TAC/DOCA surgery. **E** Fractional shortening (FS) measured at 10 days after TAC/DOCA surgery. **F** Left ventricle mass (LV mass Cor) measured at 10 days after TAC/DOCA surgery. **G** Left ventricle weight to tibia length (LV/tibia). **H** Left atria weight to tibia length (LA/tibia). **I** Lung wet/dry ratio (Lung w/d ratio). **J** Liver wet/dry ratio (Liver w/d ratio). Data are presented as mean ± SD. *p < 0.05, **p < 0.01, ***p < 0.001, ****p < 0.0001. ns: not significant**. K**–**T** HF-KO-CO (n = 8; 3 M/5F), HF-KO-CO-CA (n = 7; 3 M/4F) and HF-KO-EM-CA (n = 6; 3 M/3F). **A**–**J** One-way ANOVA with Holm-Šídák's multiple comparisons test

Because we demonstrated increased NHE1 activity in HF, and because this increase was prevented by EMPA treatment, whereby these effects were not mediated through SGLT2, we next examined whether NHE1 inhibition through the specific NHE1 inhibitor Cariporide could mimic EMPA effects on HF in SGLT2 KO mice. Firstly, we validated that aortic constriction was similar between groups (Supplementary Fig. 1H). Relative to the untreated HF-KO animals, NHE1 inhibition by Cariporide treatment prevented the development of all HF-associated parameters of diastolic and systolic dysfunction (Fig. 5A–E), hypertrophy (Fig. 5F–H) and edema of lung and liver (Fig. 5I, J). Notably, adding EMPA to the Cariporide treatment did not lead to additional protective effects (Fig. 5A–I). Thus, inhibition of NHE1 is likely an upstream cellular protection mechanism against HF by SGLT2i. Body weight was significantly decreased by Cariporide and EMPA’s effects on body weight was lost when NHE1 was inhibited by Cariporide (Supplementary Fig. 1I).

### NHE1 downregulation and NCX inhibition partly reversed PE-induced cellular hypertrophy and abolished EMPA’s protection on hypertrophy

**Fig. 6:**
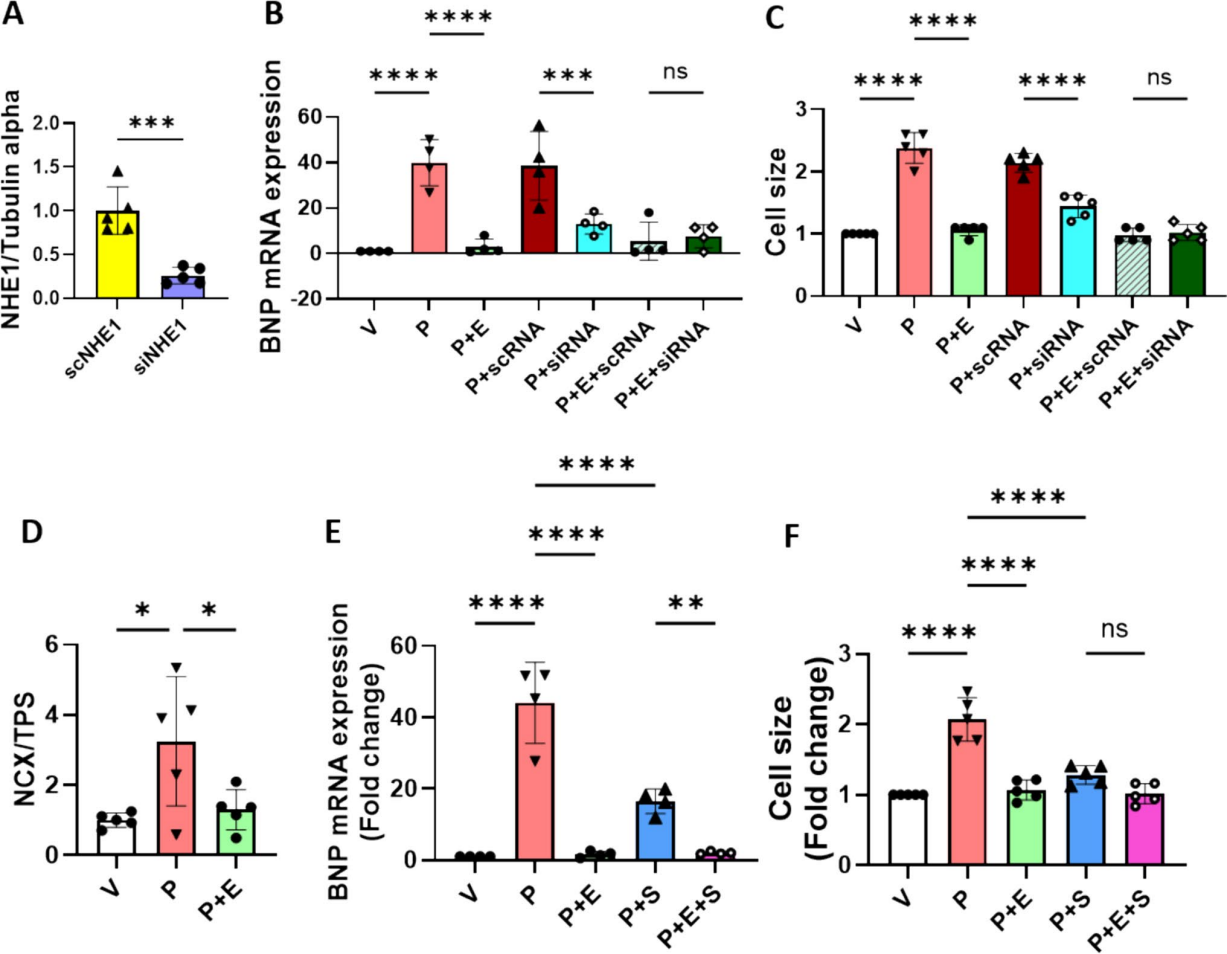
NHE1 downregulation and NCX inhibition partly reversed PE-induced cellular hypertrophy and abolished EMPA’s protection on hypertrophy. **A** Representative immunoblots and analysis of NHE1 protein in H9c2 cells with scRNA and siRNA treatment (5 individual experiments). Unpaired t test. **B** BNP mRNA expression was measured by RT-PCR after 48 h treatment under each condition. One way ANOVA with Holm-Šídák's multiple comparisons test (4 individual experiments). **C** Cell surface area was measured at 48 h. One way ANOVA with Holm-Šídák's multiple comparisons test (5 individual experiments). **D** Representative immunoblots and analysis of NCX protein in H9c2 cells with PE and PE plus EMPA treatments. One way ANOVA with Holm-Šídák's multiple comparisons test (5 individual experiments). **E** BNP mRNA expression was measured by RT-PCR after 48 h treatment under each condition. One way ANOVA with Holm-Šídák's multiple comparisons test (4 individual experiments). **F** Cell surface area was measured at 48 h. One way ANOVA with Holm-Šídák's multiple comparisons test (5 individual experiments). Data are presented as mean ± SD. *p < 0.05, **p < 0.01, ***p < 0.001, ****p < 0.0001. ns: not significant

In order to determine whether NHE1 downregulation was involved in PE-induced hypertrophy and EMPA’s protection in our model [12], we knocked down NHE1 protein by small interfering RNA. NHE1 protein expression was successfully downregulated before and after PE treatment (Fig. 6A and Supplementary Fig. 2F–H). Additionally, knock down of NHE1 prevented the PE-induced increases of BNP mRNA and cell surface area and abolished EMPA’s protection on hypertrophy (Fig. 6B, C).

To examine NCX’s role in PE-induced hypertrophy and EMPA’s protection in the same model, we check the protein level of NCX in PE and PE plus EMPA-treated hypertrophy. We found NCX protein expression was increased in PE-induced hypertrophy and was reversed by EMPA (Fig. 6D and Supplementary Fig. 2I). Furthermore, we found that NCX inhibition partly reversed PE-induced increase of BNP mRNA and cell surface area and abolished EMPA’s protection on hypertrophy (Fig. 6E, F).

### EMPA reduces HF-induced elevations in oxidative/nitrosative stress independent of SGLT2

**Fig. 7:**
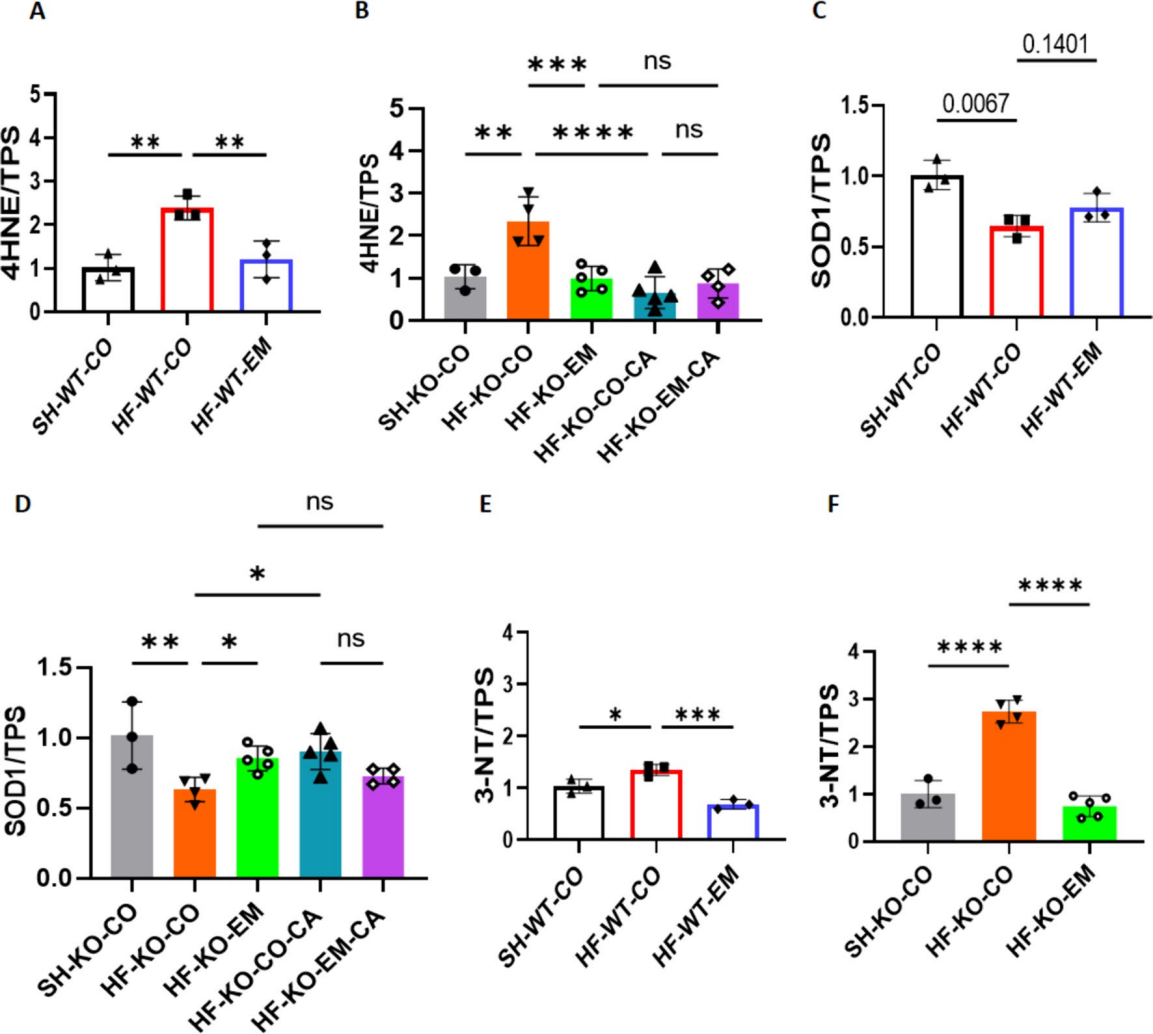
EMPA reduced HF-induced elevations oxidative/nitrosative stress independent of SGLT2. **A**, **B**, 4-hydroxynonenal protein expression/total protein staining (4-HNE/TPS). **C**, **D** Superoxide dismutase 1 protein expression/total protein staining (SOD1/TPS). **E**, **F**, 3-nitrotyrosine protein expression/total protein staining (3-NT/TPS). Data are presented as mean ± SD. *p < 0.05, **p < 0.01, ***p < 0.001, ****p < 0.0001. ns: not significant. SH-WT-CO (n = 3; 1 M/2F), SH-KO-CO (n = 3; 1 M/2F), HF-WT-CO (n = 3; 2 M/1F), HF-KO-CO (n = 4; 2 M/2F), HF-WT-EM(n = 3; 2 M/1F) and HF-KO-EM(n = 5; 2 M/3F), HF-KO-CO-CA (n = 4; 2 M/3F) and HF-KO-EM-CA (n = 5; 2 M/2F). **A**–**F** One-way ANOVA with Holm-Šídák's multiple comparisons test

Some studies have highlighted the anti-oxidative and anti-nitro-oxidative stress properties of SGLT2i [11, 58]. Overall, we observed increased 4-HNE and decreased SOD1 as markers of oxidative stress, and increased 3-nitrotyrosine 3-NT as marker of increased peroxinitrite in HF hearts, respectively, whereby EMPA treatment reduced almost all of these markers. Both HF and EMPA effects were independent of the presence of SGLT2. The NHE1 inhibitor Cariporide also reduced these markers of oxidative stress, on top of which EMPA was unable to further reduce oxidative stress markers (Fig. 7A–F**,** Supplementary Fig. 2J–N). Our data thus demonstrate that oxidative and nitrosative stress are strongly induced in our TAC/DOCA-HF model, and EMPA treatment is able to prevent these increases, independent of the presence of SGLT2, and in a similar fashion as NHE1 inhibition.

### Nitric oxide synthase (NOS) inhibition abolishes EMPA-induced HF protection

The presented data so far, indicate that NHE1 inhibition is a possible upstream effector of the protective effects of EMPA. Additionally, assuming EMPA protects through the NHE-NO pathway, insufficient NO has been suggested as a final downstream effect of EMPA [11, 18]. The decreased NO levels in HF cardiac tissue are likely a consequence of heightened oxidative stress, which subsequently interacts with NO to generate peroxynitrite. Our HF-model shows that these two processes are operative, as indicated by the oxidative/nitrosative stress biomarkers 4-HNE and 3-NT, which are mitigated by EMPA, indicative that EMPA may restore cardiac NO levels (Fig. 7).

**Fig. 8:**
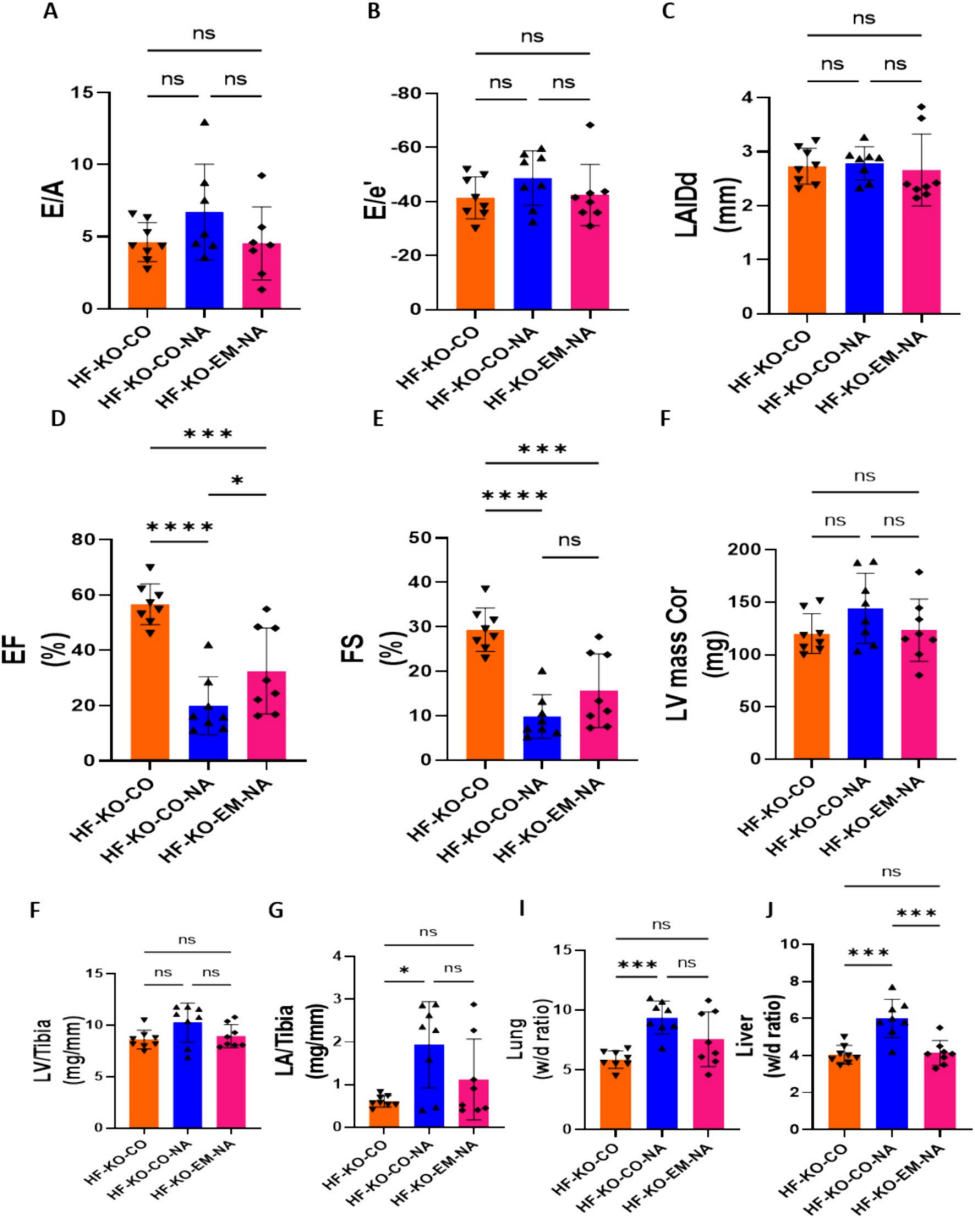
Nitric oxide synthase (NOS) inhibition abolished HF protection by EMPA. **A** The ratio of early diastolic mitral inflow velocity to late diastolic mitral inflow velocity (E/A ratio) measured at 10 days after TAC/DOCA surgery. **B** The ratio of early diastolic mitral inflow velocity to early diastolic mitral annulus velocity (E/e' ratio) measured at 10 days after TAC/DOCA surgery. **C** Left atria internal diameter end diastole (LAIDd) measured at 10 days after TAC/DOCA surgery. **D** Ejection fraction (EF) measured at 10 days after TAC/DOCA surgery. **E** Fractional shortening (FS) measured at 10 days after TAC/DOCA surgery. **F** Left ventricle mass (LV mass Cor) measured at 10 days after TAC/DOCA surgery. **G** Left ventricle weight to tibia length(LV/tibia). **H**, Left atria weight to tibia length (LA/tibia). **I**, Lung wet/dry ratio (Lung w/d ratio). **J**, Liver wet/dry ratio (Liver w/d ratio). Data are presented as mean ± SD. * p < 0.05, *** p < 0.001, **** p < 0.0001. ns: not significant. A-K: HF-KO-CO (n = 8; 3 M/5F), HF-KO-CO-NA (n = 8; 4 M/4F) and HF-KO-EM-NA (n = 8; 4 M/4F). **A-J**: One-way ANOVA with Holm-Šídák's multiple comparisons test

If improvement in NO is indeed instrumental to EMPA’s protection, interventions that inhibit NO synthesis and lead to less NO should worsen HF, and most importantly, should prevent protection by EMPA. We tested this hypothesis by adding L-NAME, a nitric oxide synthase (NOS) inhibitor, to the drinking water of TAC/DOCA treated animals. An additional group of mice were treated with EMPA on top of NOS inhibition by L-NAME. First, we showed that the aortic constriction generated a similar insult on the aorta in these two groups (Supplementary Fig. 1 J). Compared to the HF-KO mice administered with control chow (HF-KO-CO group), L-NAME treatment worsened some HF parameters, but not all. Most importantly, in the presence of L-NAME, EMPA treatment was no longer able to improve any of the HF parameters as compared to HF group that was not treated with EMPA and L-NAME (Fig. 8A–I). This indicates that EMPA’s downstream effect may indeed be an increased presence of NO in cardiac tissue. Note that, although EMPA did not provide protection after L-NAME application when compared with HF-KO-CO group, EMPA was protective against L-NAME-induced liver edema and slightly increased EF (comparing HF-KO-CO-NA group with HF-KO-EM-NA group) (Fig. 8J and D). Body weight was unaffected by L-NAME and EMPA reduced body weight (Supplementary Fig. 1 K). Our data, therefore, support the contention that a major part of the protective effect of EMPA against HF may be related to improvement of NO homeostasis in the heart.

### Plasma levels of EMPA

The 300 mg EMPA/kg chow resulted in a plasma EMPA concentration of 0.29 ± 0.10 µM (Supplementary Table 1).

## Discussion

This study shows that, (1) whole body knockout of SGLT2 alone does not attenuate the development of HF, except for a mild protection against systolic dysfunction, (2) EMPA protects against all HF parameters, similarly in WT and SGLT2 KO animals, (3) NHE1 inhibition mimics EMPA by normalizing or mitigating all HF-related parameters. Adding EMPA to Cariporide induced specific NHE1 inhibition has no additive effects, (4) Nitric oxide synthase inhibition aggravates HF induced by TAC/DOCA, and fully antagonizes HF protection by EMPA. Thus, we herein demonstrate that the profound beneficial effects of EMPA on HF are independent of SGLT2 and its whole-body effects, and are likely mediated through NHE1 inhibition and improved NO signaling through the NHE1/NCX/oxidative stress/CamKII/NO pathway.

### SGLT2 inhibition does not mediate HF protection by EMPA

SGLT2i were developed for diabetic patients to target and inhibit SGLT2 in the kidney, thereby preventing glucose reabsorption and causing glucose excretion [74]. Thus, these drugs may induce a nutrient deprivation status of the body, that under fasting conditions, can even result in impactful increases (> 0.1 mM rise) in levels of ketone bodies [57]. These whole body metabolic effects due to SGLT2 inhibition in the kidney have been proposed by several researchers as the main mechanism through which SGLT2i provide cardioprotection [9, 23, 50, 56, 57]. We now show that SGLT2i protection against HF in our non-ischemic TAC/DOCA murine model of HF is also operative in the absence of SGLT2. Previously, we already demonstrated that SGLT2i-induced protection against acute myocardial ischemia is equally present in WT and SGLT2 KO animals [14]. Recently, it has also been shown that the SGLT2i dapagliflozin (DAPA) attenuates HF in SGLT2 deficient animals [69]. Although, in that latter study, C57BL/6J animals were used which lack the mitochondrial enzyme nicotinamide nucleotide transhydrogenase (NNT) known to play a crucial role in HF and present in humans, and suggesting its use in cardiovascular research problematic [47]. The present work demonstrates that SGLT2i protection against HF is independent of SGLT2 in animals carrying this important mitochondrial enzyme. It is thus unlikely that SGLT2i effects are mediated through these whole body metabolic effects of glycosuria. Our present study supports the notion that off-target effects of SGLT2i are responsible for the beneficial effects of EMPA. We and others have previously shown that SGLT2i can inhibit and interact with the NHE1 and lead to decreased cytoplasmatic sodium and calcium concentrations [4, 10, 25, 33]. We here have provided evidence that cardiac NHE1 inhibition is upstream of the protective pathway.

It is, however, still possible that the protective effects are mediated through other extra-cardiac mechanisms of SGLT2i, for example through inhibition of NHE1 present in most cells of the body, or NHE3 in the proximal tubule of the kidney, for which evidence has been reported [8]. Further research with for example specific NHE1/3 knock-out models or tissue/cell-specific NHE KO using Cre-Lox system are needed to explore these alternative explanations.

### Inhibition of NHE1 plays an important initiator role in the beneficial effects of EMPA

Although SGLT2i can inhibit NHE1 activitiy in isolated cells of various origin, (such as cardiomyocytes [4, 15, 30, 33, 37, 52, 62, 63, 75], endothelial cells [10, 25, 65], fibroblasts [72], platelets [60] and immune cells [34]) the interaction between SGLT2i and NHE1 at the cellular level has not always been observed [17]. Although methodological differences between these studies were suggested for the cellular experiments, subsequent work demonstrated that these differences were unable to explain the differences [75]*.* However, more recent work demonstrated that the use of protease XIV in cell isolation procedures could explain the differences between the cellular studies. Protease XIV abrogated the interaction between SGLT2i and NHE1, but not between cariporide and NHE1, also indicating that the binding site of cariporide to NHE1 is different from the binding site of EMPA to NHE1 [13]. The interaction between SGLT2i and NHE1 is also not always observed at the level of the healthy intact heart [17, 66]. It seems that under pathological conditions such as cardiac ischemia or disturbances in cardiac ion homeostasis the interaction becomes most obvious, also in the intact heart [52, 66]. Previous preclinical models have demonstrated the crucial causal role of NHE1 activity in HF [2, 3, 19, 35, 46]. However, the development of clinically-applicable NHE1 inhibitors came to an abrupt halt when these inhibitors were associated with unexpected cerebrovascular events in the setting of acute myocardial infarction in coronary-artery bypass grafting (CABG) and percutaneous transluminal coronary angioplasty (PTCA) patients, despite achievement of its primary endpoint (reduction in infarct size) [42]. As a consequence, NHE1 inhibitors have never been clinically tested for efficiency in the setting of HF. It should thereby be noted that the cerebrovascular events in patients shortly treated with high dosage of the NHE1 inhibitor all happened after cessation of inhibitor treatment, not during the treatment (personal communication Dr. R Mentzer) [42]. It is possible, although speculative, that stroke occurred because of the sudden and total withdrawal of the NHE1 inhibitor. This could have evoked a rebound effect on platelet activation. The notion that stroke did not occur in patients that were on-treatment makes it unlikely that these cerebrovascular events will occur in patients that remain on treatment, as is commonly the case with SGLT2i therapy. In this respect, it is interesting to note that in the large EMPA-REG outcome study, there was a numeric difference in the hazard ratio for stroke, primarily caused by patients that stopped using the study drug [73]. Thus, it is very well possible that SGLT2i therapy in humans proves beneficial in the prevention of HF by NHE1 inhibition. To assess the potential beneficial or harmful effects of NHE1 inhibition in HF patients, further experiments on preclinical, comorbidity models will be needed [20, 21].

In the current study, the increased NHE1 activity with HF was subsequently decreased by EMPA treatment. Notably, NHE1 activity was reduced in isolated cardiomyocytes from HF-EMPA treated animals in the absence of EMPA. This finding cannot be used as argument that EMPA effects on NHE1 are thus indirect***,*** because it is known that NHE1 activity is also post-translationally regulated, and that the presence of EMPA in vivo will attenuate this negative feedback loop on increasing NHE1 activity through post-translational regulation [11, 18]. Cariporide almost completely mimics the effects of EMPA, thus providing a pharmacological NHE1 knock-out model with loss of any remaining protective actions of EMPA on top of Cariporide. NHE1 inhibition by EMPA may indeed be a main upstream mechanism of HF protection. However, the ultimate proof for such mechanism will be the generation of animals with a specific mutation of the SGLT2i-binding site within NHE1, with maintenance of the normal function of NHE1, and subjecting these animals to HF and SGLT2i treatment. This approach is at this moment not feasible because the specific binding site for EMPA is currently unknown. However, a recent study shows that a novel compound derived from a classic SGLT2i (and modified to have much less SGLT2 inhibition with improved NHE1 binding and inhibition), provides even stronger protection than the classic SGLT2i [71].

### Improving NO homeostasis as important end-effector in EMPA’s protection

Depletion of NO in cardiac tissue has long been known as an important driver of HF [32, 59]. Therapies directed at improving cardiac NO content and its upstream or downstream factors, such as NO donors, soluble guanylate cyclase (sGC) stimulators or phosphodiesterase 5 (PDE5) inhibitors, have not been very successful clinically, especially in treating HFpEF patients [59]. One of the major reasons why NO is lowered during HF is the increase in oxidative stress and inflammatory mediators, with ROS quickly reacting with NO to produce the detrimental peroxinitrite [22, 51]. The increase in 3-NT in our untreated HF hearts is indicative of increases in peroxinitrite, knowing that 3-NT is, at least partly, caused by peroxinitrite. Therefore, direct targeting of the initial mechanism that drives the depletion of NO in heart failure, i.e. the reduction in oxidative stress by SGLT2i as shown in the current report, may be a clinically-effective avenue to treat low cardiac NO content. We and others have shown that cytokines increase ROS production in human endothelial cells and concomitantly decrease NO production, and that both processes are mitigated by SGLT2i [31, 64, 65]. The improved NO in endothelial cells was subsequently shown to activate cellular signaling in cardiomyocytes to increase their function [31]. Our current observations that EMPA’s protective actions are completely abrogated when NO synthesis is blocked by high dosage of L-NAME are in support of the importance of maintaining a critical cardiac NO content and that, at least partly, one of the end-effector mechanisms of SGLT2i may be the improvement of cardiac NO content.

### The involvement of the NHE1/NCX/ROS/CamKII/NO pathway in EMPA’s protection

It is now recognized that especially HFpEF is driven by various co-morbidities, such as metabolic syndrome, obesity, chronic low grade inflammation, ageing and hypertension [59]. Many of these co-morbidities can directly increase intracellular sodium loading of cardiac cells [11, 18]. For example, high glucose [4], TNF-α [65] and mechanical stress [54] were shown to increase cardiac intracellular sodium concentration. These increases in sodium can be facilitated by increased activity of NHE1, the development of the late Na^+^ current through the Na_v_1.5 sodium channel, the reversal of the NCX or impairment of the Na^+^/K^+^-ATPase (NKA) [52]. The increased NCX expression in our HF model matches with previous finding in failing hearts and is indicative of disturbances in intracellular sodium and/or calcium [5, 29, 61]. Interestingly, many of these plasma sodium loaders have shown potential to be modulated by SGLT2i. EMPA has also been shown to inhibit the late Na^+^ current, albeit probably indirectly [26, 45, 53], or the NCX [37, 68]. The increase in Na^+^, and the consequently increase in Ca^2+^ can then result in oxidative stress through Ca^2+^-activated NOX activation. We recently demonstrated that increases in intracellular calcium in endothelial cells generated ROS, which was reduced by EMPA in human endothelial cells through the NHE1/PKC/NOX pathway [38]. The increased ROS, together with calcium, can then facilitate CamKII activation, a crucial driver of cardiac remodelling [36]. Our data is in full support of such a pathway, showing that HF in our short-term hypertensive model increased cardiac NHE1 activity, NCX expression, oxidative stress, and CamKII phosphorylation, and that EMPA treatment prevents all these cellular changes. In addition, our cellular studies with NHE1 knockdown experiments and NCX inhibition studies in a cellular hypertrophy model, build further proof for the critical involvement of NHE1 and NCX in EMPA’s protective mechanism. These ionic disturbances that can be corrected by SGLT2i may also underlie SGLT2i reported antiarrhythmic effects for especially atrial cells [49], although care should be taken that these effects of SGLT2i are studied at therapeutic relevant doses of SGLT2i [16].

### Methodological limitations

Although EMPA applied to isolated cardiac myocytes did have the ionic effects mentioned, it cannot be excluded that the protective effects are mediated through other extra-cardiac mechanisms of SGLT2i, for example through inhibition of NHE1 present in most cells of the body, or NHE3 in the proximal tubule of the kidney, for which evidence has been reported [8]. One such example was the demonstration of NHE1 inhibition in B lymphocytes by EMPA, which inhibition prevented excessive loss of these B cell through impairment of autophagy and improved cardiac function and reduced infarct size following myocardial infarction [70].

Furthermore, although human studies show that SGLT2 was not expressed in the myocardium, there is disagreement in the field with regards to the presence or absence of SGLT2 in cardiac myocytes, as SGLT2 mRNA has occassionally been reported in left ventricular myocytes from human hearts [41]. We have used a short-term model of moderate HF development through the combination of TAC with a DOCA pellet. Previous work demonstrated that the pressure-overload induced hypertrophy by the TAC procedure, sensitizes the heart to mineralocortocoid overload, thereby accelerating the transition to a HFpEF [44]. We choose specifically for this type of HFpEF murine model since the popular high-fat diet HFpEF model, in combination with NO inhibition, does not lend itself for studying SGLT2i mechanisms. Such models exhibit large hyperglycemia and permanent inhibition of endothelial NO production. In such model, SGLT2i will have a large impact on plasma glucose levels (probably lowering plasma glucose by > 5 mM) but cannot improve NO homeostasis. However, SGLT2i use in human clinical trials only reduce plasma glucose levels by ~ 0.5 mM with the potential of increasing NO through reductions in oxidative stress. Thus, the high fat/L-NAME HFpEF model would be inappropriate for studying the underlying mechanisms of SGLT2i. However, in our hands the TAC/DOCA-insult did reduce ejection fraction, although only mildly, generating a HFmrEF rather than a HFpEF phenotype. Possibly the use of C57BL/6N mice instead of FVB/NJ mice [44] or C57BL/6 J mice [43] is causing this different outcome with the TAC/DOCA-insult. Nevertheless, we use a murine model that represents early HF, the target cohort of human patients with HF.

Although it may seem that we used a high dosing of EMPA through application of 300 mg EMPA/kg chow, measurements of plasma levels of EMPA demonstrated this not to be the case. The averaged EMPA concentration of 0.3 µM determined early after feeding are similarly to the Cmax values of EMPA determined early after EMPA administration in humans (0.3–0.6 µM) [27, 28]**.** Therefore, clinically relevant concentrations of EMPA were studied in our animal model of HF.

Both EMPA and cariporide treatment resulted in a body weight reduction of approximately 5%. It could therefore be argued that protective effects of these treatment are mediated through body weight reductions. However, although similar body weight reductions were observed in clinical trials with SGLT2i, no associations between the cardiovascular beneficial effects of SGLT2i and body weight reduction were present [1]. That the reduction in body weight could mediate the SGLT2i cardiovascular effects is also unlikely because of the early appearance of SGLT2i’s beneficial cardiac effects (within weeks to months) that have never been observed for body weight reduction interventions. In addition, the use of L-NAME in our animal study also resulted in a similar body weight reduction, whereas cardiac function deteriorated. Thus, although we cannot exclude the possibility that reductions in body weight contributed to EMPA’s protective effects described in this study, current information makes this an unlikely explanation.

Recently, it has been shown that SGLT2i decreased the formation of uremic toxins by the microbiome, independent of SGLT2, and that these uremic toxins induced detrimental remodeling of the heart [7]. Thus, we cannot exclude that these SGLT2-independent effects also play a role in our HF model. Further research is awaited to examine whether this mechanism is also operative in our short-term model of HF.

In the current study we have applied a non-ischemic TAC/DOCA model of HF. It is possible that results could differ from those obtained in an ischemic, LAD occlusion, model of HF. However, we already demonstrated that EMPA’s protection against HF following infarction following an cardiac ischemic insult is also independent of SGLT2 [14]. Secondly, a very recent publication employing a chronic CAD model of HF also demonstrated that SGLT2i protection against HF was independent of SGLT2 [69]. However, whether SGLT2i protection in these two models of cardiac diseases is also mediated through NHE1-NO axis was not, and is awaiting further research.

## Conclusion

We here provide support of the hypothesis that both NHE1 inhibition and improved NO homeostasis, are playing an important role in the protective mechanisms of the SGLT2i EMPA in an early model of HF, whereas the role of the SGLT2 protein in the beneficial effects of SGLT2i is minimal. NHE1 inhibitors are potentially promising agents in the prevention of human HF.

## Supplementary Information

Below is the link to the electronic supplementary material.

Supplementary file1 (DOCX 4257 KB)

## Data Availability

The authors declare that the datasets used and/or analyzed in the current study are available from the corresponding author upon reasonable request.
