# Supplementary material for: Empagliflozin prevents heart failure through inhibition of the NHE1-NO pathway, independent of SGLT2
Source: Basic Res Cardiol. 2024 Jul 24;119(5):751–72. doi: 10.1007/s00395-024-01067-9 (PMC11461573; doi:10.1007/s00395-024-01067-9)
Supplement: Supplementary file 1 — Supplementary file1 (DOCX 4257 KB) [file 395_2024_1067_MOESM1_ESM.docx]

**Supplementary Figure 1**

**
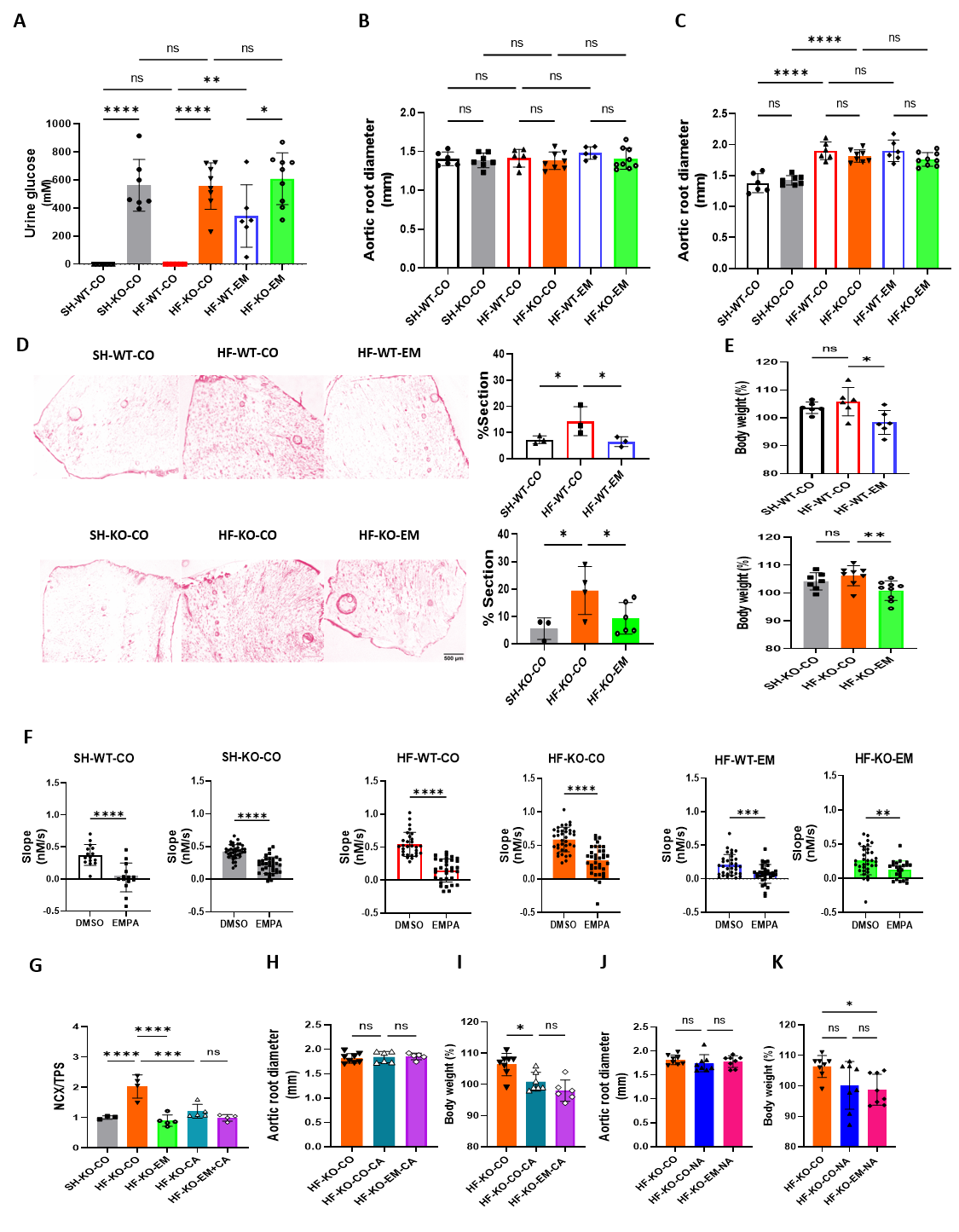
**

**Supplementary Figure 1 A**. Urine glucose detected at 10-12 days after TAC/DOCA surgery for series #1. **B**, Aortic root diameter measured by echocardiography 2 days before TAC/DOCA surgery for series #1. **C**, Aortic root diameter measured by echocardiography 10 days before TAC/DOCA surgery C **D**, Cardiac fibrosis, Sirius red images were semi-automated analyzed for the percentage fibrosis area in left ventricle free wall (% Section) for series #1. **E**, Body weight on day 10 normalized to day 0 for series #. **F**, Acute EMPA effects on NHE1 activity of cardiomyocytes isolated from hearts of series #1. **G** Aortic root diameter measured by echocardiography 10 days before TAC/DOCA surgery for series #2. **H**, Body weight on day 10 normalized to day 0 for series #2. **I**, Aortic root diameter measured by echocardiography 10 days before TAC/DOCA surgery for series #3.**J**, Body weight at end point normalized to day 0 for series #3. Data are presented as mean ± SD. * p < 0.05, ** p < 0.01, **** p < 0.0001. **A-C** and **F**: SH-WT-CO (n=6; 3M, 3F), SH-KO-CO (n=7; 4M/3F), HF-WT-CO (n=6; 3M/3F), HF-KO-CO (n=8; 3M/5F), HF-WT-EM (n=6; 3 M/3F) and HF-KO-EM(n=9; 4M/5F). **D**: SH-WT-CO (n=3; 1M/2F), SH-KO-CO (n=3; 1M/2F), HF-WT-CO(n=3; 2M/1F), HF-KO-CO (n=4; 2M/2F), HF-WT-EM(n=3; 2 M/1F) and HF-KO-EM(n=5; 2M/3F). **E**: SH-WT-CO (15/14 cells from 3 mice, 2M/1F), SH-KO-CO (39/34 cells from 4 mice, 3M/1F), HF-WT-CO (29/32 cells from 3 mice, 1M/2F), HF-KO-CO (37/36 cells from 4 mice, 1M/3F), HF-WT-EM(33/32 cells from 3 mice, 1M/2F) and HF-KO-EM (34/23 cells from 3 mice, 1M/2F). **G-H**: HF-KO-CO (n=8; 3M/5F), HF-KO-CO-CA (n=7; 3M/4F) and HF-KO-EM-CA (n=6; 3M/3F). **I-J**: HF-KO-CO (n=8; 3M/5F), HF-KO-CO-NA ( n=8; 4M/4F) and HF-KO-EM-NA (n=8; 4M/4F). **A-C** and **F-I**: One-way ANOVA with Holm-Šídák's multiple comparisons test. **D**: One-way ANOVA with uncorrected Fisher’s LSD.

**
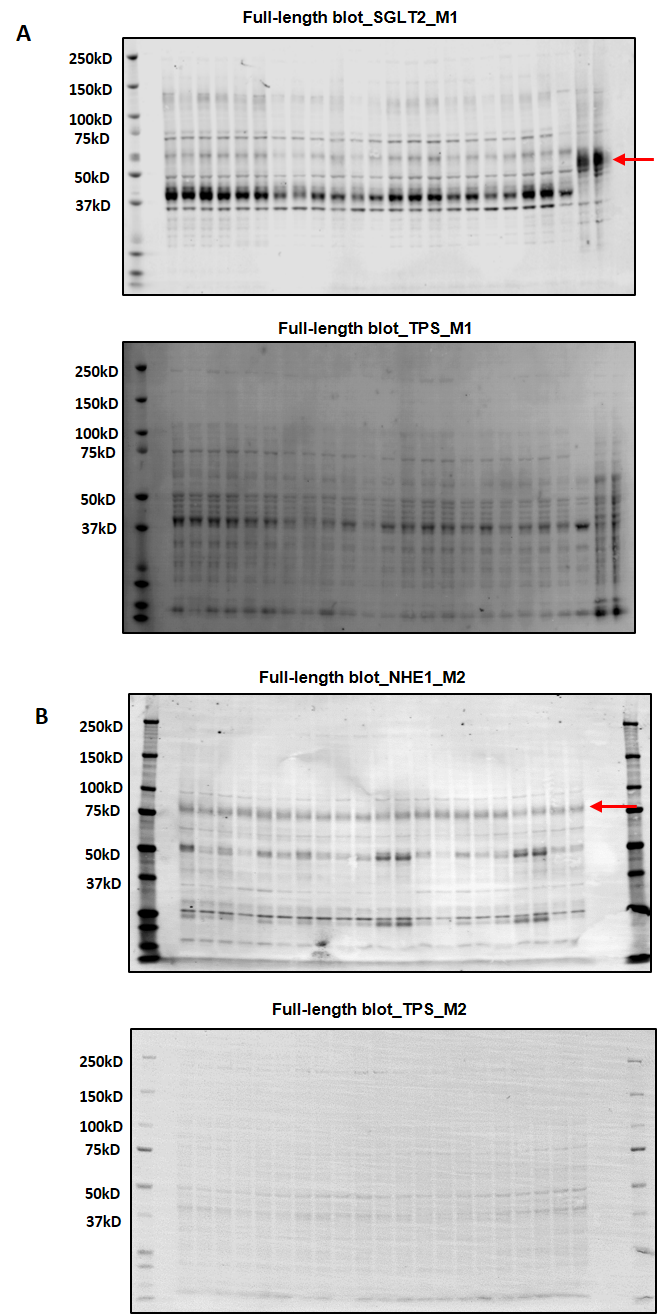
Supplementary Figure 2** the membranes for western blot.

In Membrane 1, from Lane 1 to 3: SH-WT-CO; 4 to 6: SH-KO-CO; 7 to 9: HF-WT-CO; 10 to 13: HF-KO-CO; 14 to 16: HF-WT-EM; 17 to 21: HF-KO-EM ; 22 to 23: WT-Kidney

In Membrane 2, from Lane 1 to 3: SH-WT-CO; 4 to 6: HF-WT-CO; 7 to 9: HF-WT-EM; 10 to 13: SH-KO-CO; 14 to 16: HF-KO-CO; 17 to 21: HF-KO-EM

TPS: Total protein staining


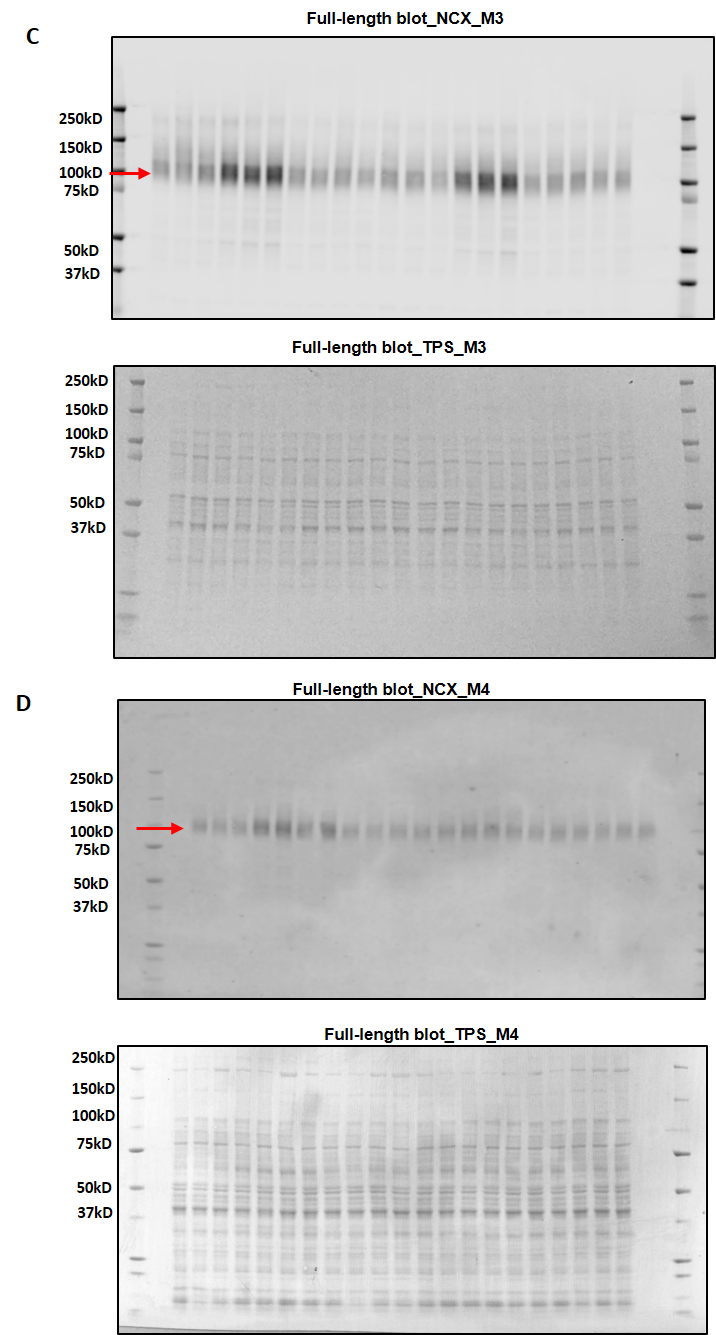


In Membrane 3, from Lane 1 to 3: SH-WT-CO; 4 to 6: HF-WT-CO; 7 to 9: HF-WT-EM.

In Membrane 4, from Lane 1 to 3: SH-KO-CO; 4 to 7: HF-KO-CO; 8 to 12: HF-KO-EM; 13 to 17: HF-KO-CO-CA; 18 to 21: HF-KO-EM-CA.

TPS: Total protein staining


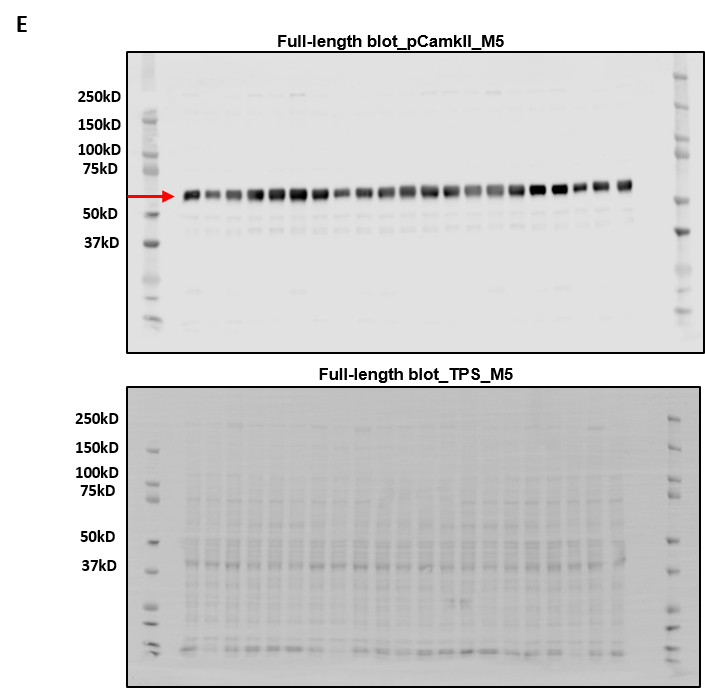


In Membrane 5, from Lane 1 to 3: SH-KO-CO; 4 to 7: HF-KO-CO; 8 to 12: HF-KO-EM; 13 to 15: SH-WT-CO; 16 to 18: HF-WT-CO;19-21: HF-WT-EM.

TPS: Total protein staining


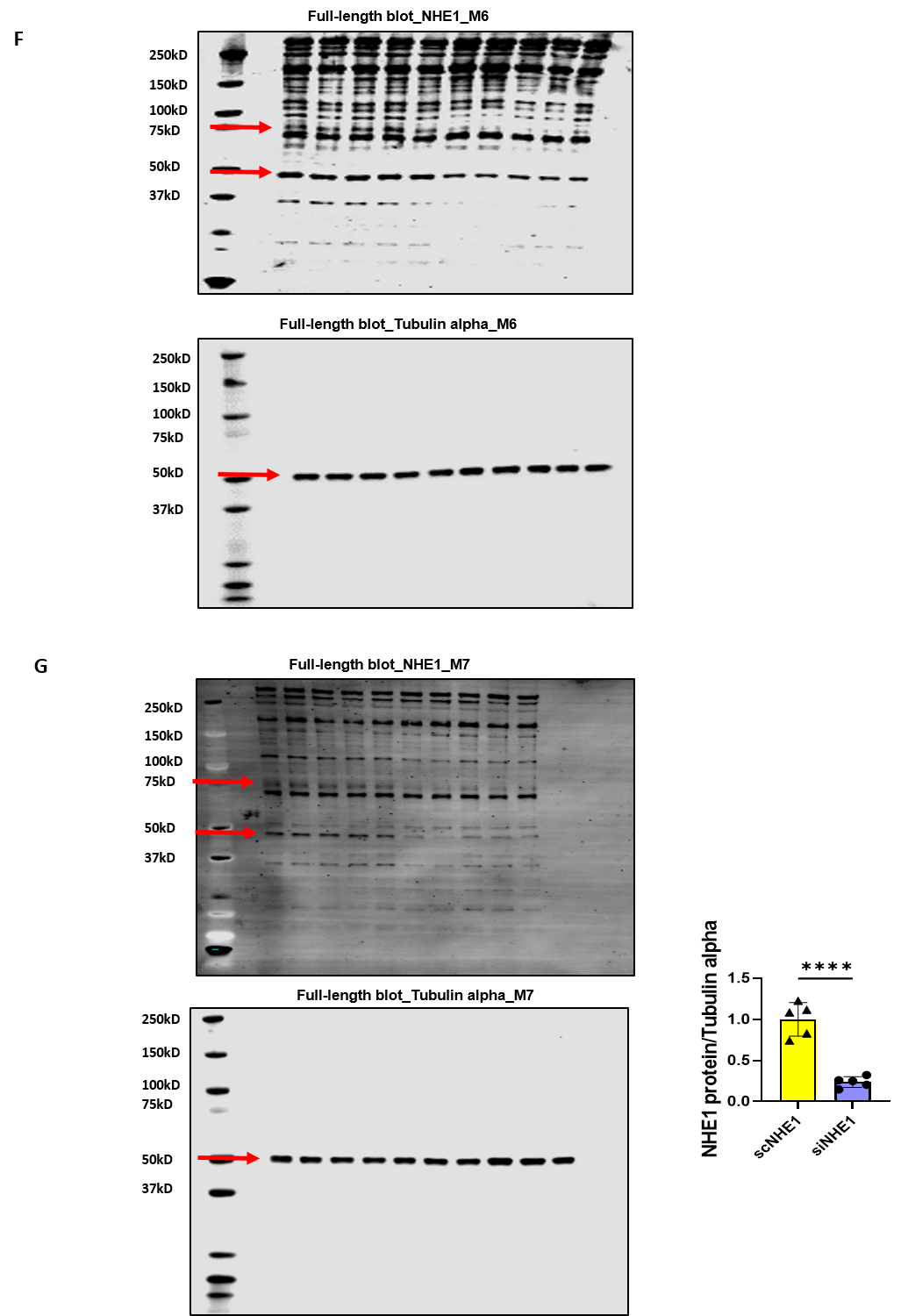


In Membrane 6, from Lane 1 to 5: P+scRNA; 6 to 10: P+siRNA.

In Membrane 7, from Lane 1 to 5: P+scRNA; 6 to 10: P+siRNA.

TPS: Total protein staining


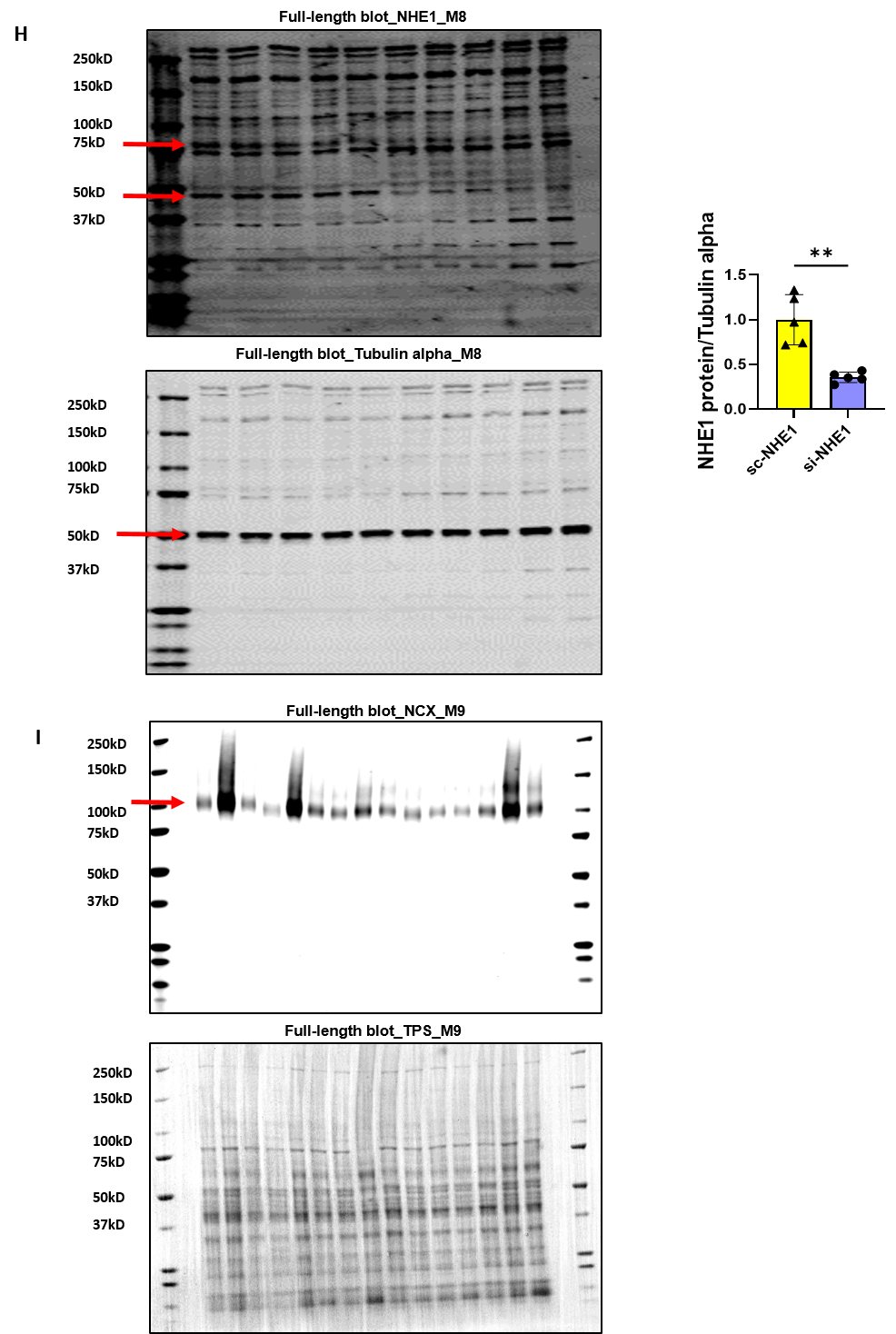


In Membrane 8, from Lane 1 to 5: P+E+scRNA; 6 to 10: P+E+siRNA.

In Membrane 9, from Lane 1 to 3: V, P and P+E; 4 to 6: V, P and P+E; 7 to 9: V, P and P+E; 10 to 12: V, P and P+E; 13 to 15: V, P and P+E.

TPS: Total protein staining


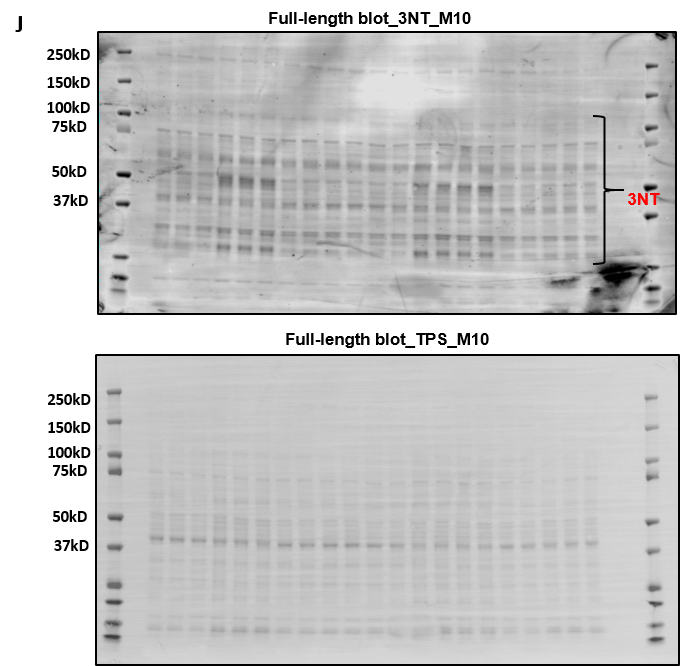


In Membrane 10, from Lane 1 to 3: SH-WT-CO; 4 to 6: HF-WT-CO; 7 to 9: HF-WT-EM; 10 to 13: SH-KO-CO; 14 to 16: HF-KO-CO; 17 to 21: HF-KO-EM.

TPS: Total protein staining


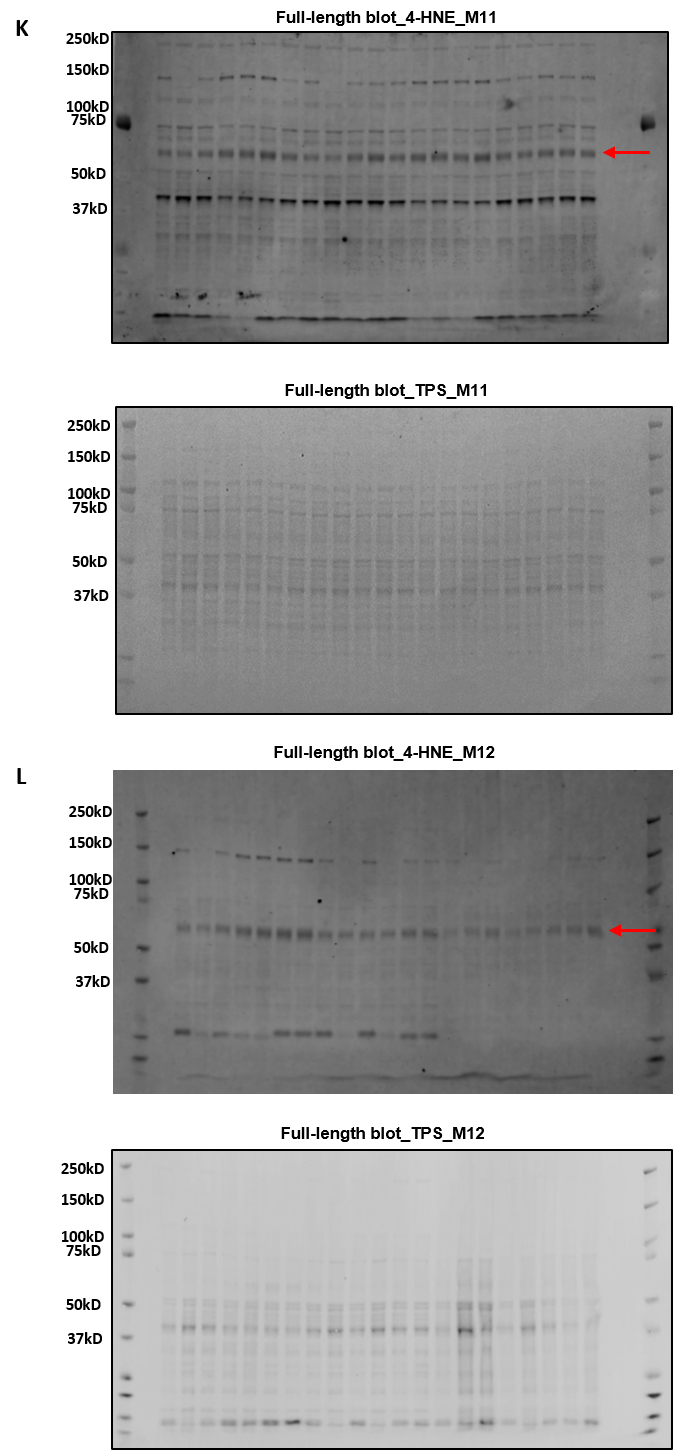


In Membrane 11, from Lane 1 to 3: SH-WT-CO; 4 to 6: HF-WT-CO; 7 to 9: HF-WT-EM.

In Membrane 12, from Lane 1 to 3: SH-KO-CO; 4 to 7: HF-KO-CO; 8 to 12: HF-KO-EM; 13 to 17: HF-KO-CO-CA; 18 to 21: HF-KO-EM-CA.

TPS: Total protein staining


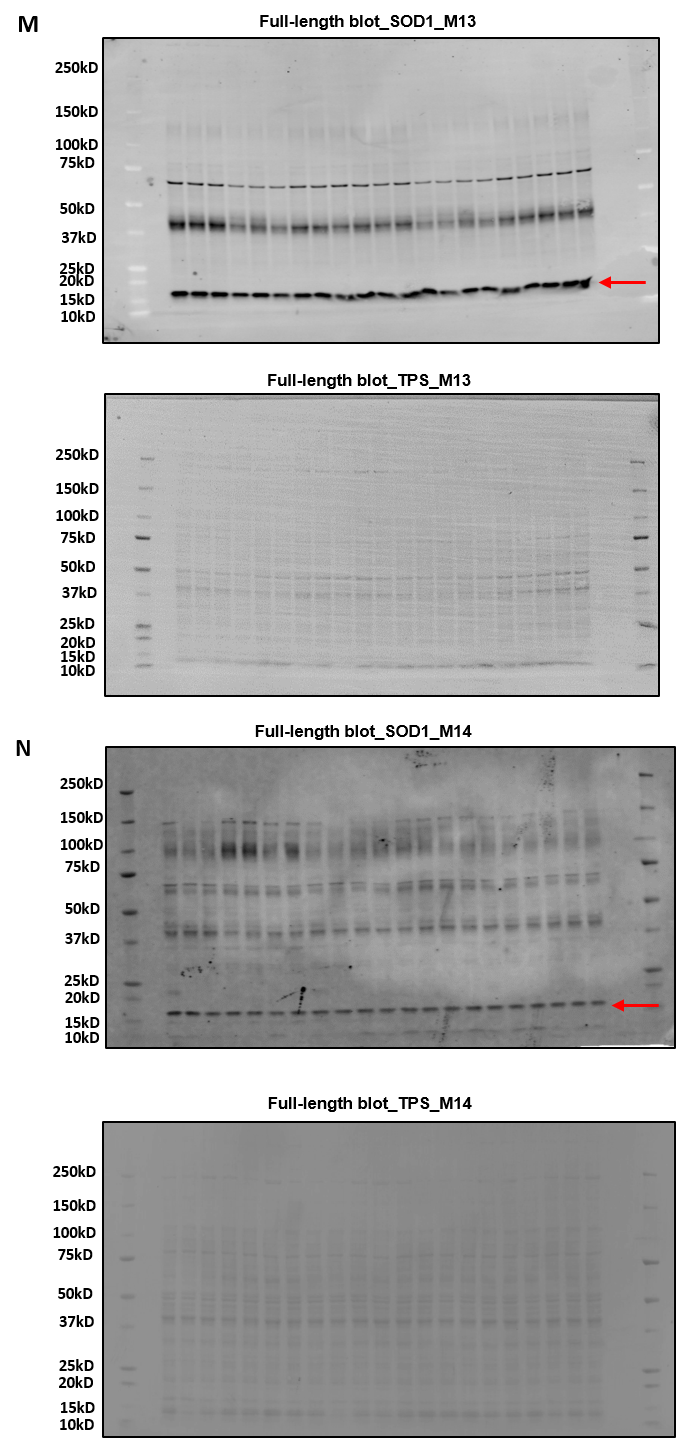


In Membrane 13, from Lane 1 to 3: SH-WT-CO; 4 to 6: HF-WT-CO; 7 to 9: HF-WT-EM.

In Membrane 14, from Lane 1 to 3: SH-KO-CO; 4 to 7: HF-KO-CO; 8 to 12: HF-KO-EM; 13 to 17: HF-KO-CO-CA; 18 to 21: HF-KO-EM-CA.

TPS: Total protein staining

**Supplementary table 1**

| Animal | Genotyping | Gender | EMPA plasma  concentration (μM) | Mean(μM) | SD(μM) |
| --- | --- | --- | --- | --- | --- |
| Mouse 1 | WT | M | 0,25 | 0,29 | 0,10 |
| Mouse 2 | KO | M | 0,14 |  |  |
| Mouse 3 | KO | M | 0,33 |  |  |
| Mouse 4 | KO | M | 0,24 |  |  |
| Mouse 5 | KO | M | 0,32 |  |  |
| Mouse 6 | KO | M | 0,43 |  |  |

**Supplementary table 1.** 6 male mice(1 WT, 5 KO) were subjected to the TAC/DOCA insult and treated them with EMPA-enriched chow. At 6-7 days after EMPA treatment, animals were anesthetized and blood drawn from the cannulated carotid artery between 9-11 AM in the morning for plasma EMPA concentration determination by the France company Eurofins ADME BIOANALYSES .
